# Supplementary material for: De novo design of protein minibinder agonists of TLR3
Source: Nat Commun. 2025 Jan 31;16:1234. doi: 10.1038/s41467-025-56369-w (PMC11785957; doi:10.1038/s41467-025-56369-w)
Supplement: Supplementary file 1 — Supplementary Information [file 41467_2025_56369_MOESM1_ESM.pdf]

Supplementary Information

De novo design of protein minibinder agonists of TLR3

Chloe S. Adams[1,2], Hyojin Kim[3], Abigail E. Burtner[1,2], Dong Sun Lee[3], Craig Dobbins[1,2], Cameron Criswell[1,2], Brian Coventry[1,2,4], Adri Tran-Pearson[1,2], Ho Min Kim[3,5,*], and Neil P. King[1,2,*]

[1]Institute for Protein Design, University of Washington, Seattle, WA, 98195 USA.
[2]Department of Biochemistry, University of Washington, Seattle, WA, 98195 USA.
[3]Center for Biomolecular & Cellular Structure, Institute for Basic Science (IBS), Daejeon 34126, South Korea.
[4]Howard Hughes Medical Institute, University of Washington, Seattle, WA, USA.
[5]Department of Biological Sciences, Korea Advanced Institute of Science and Technology (KAIST), Daejeon 34141, South Korea.

*Correspondence to Ho Min Kim and Neil P. King. Email: hm_kim@kaist.ac.kr, neilking@uw.edu

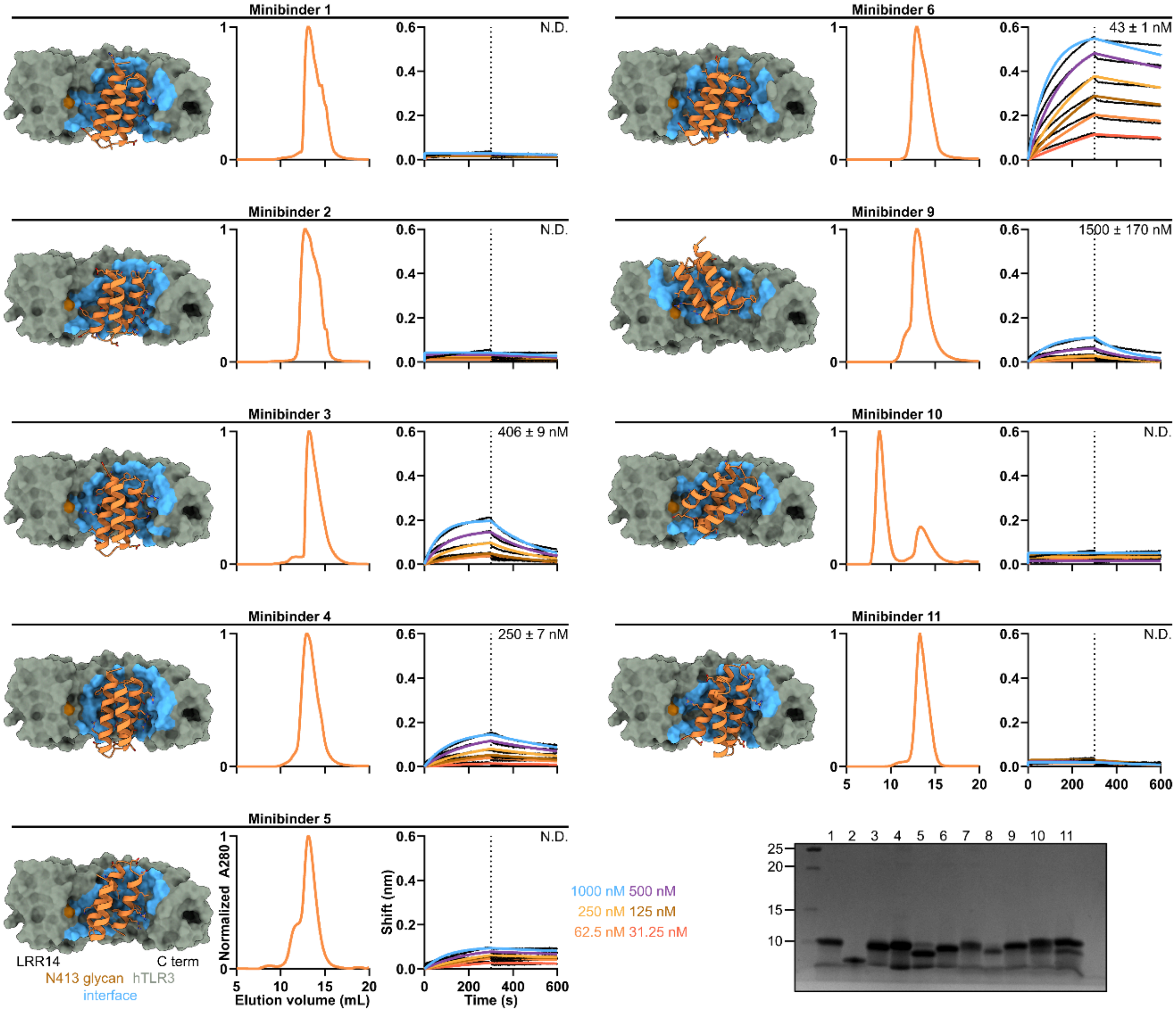

Supplementary Fig. 1: Experimental characterization of hits obtained by yeast display. From left to right, design model, SEC, and BLI for each minibinder. KD values are listed; N.D. indicates poor fit or no binding. Bottom, reducing SDS-PAGE of minibinders 1-11. The protein ladder (left) is labeled in kDa.

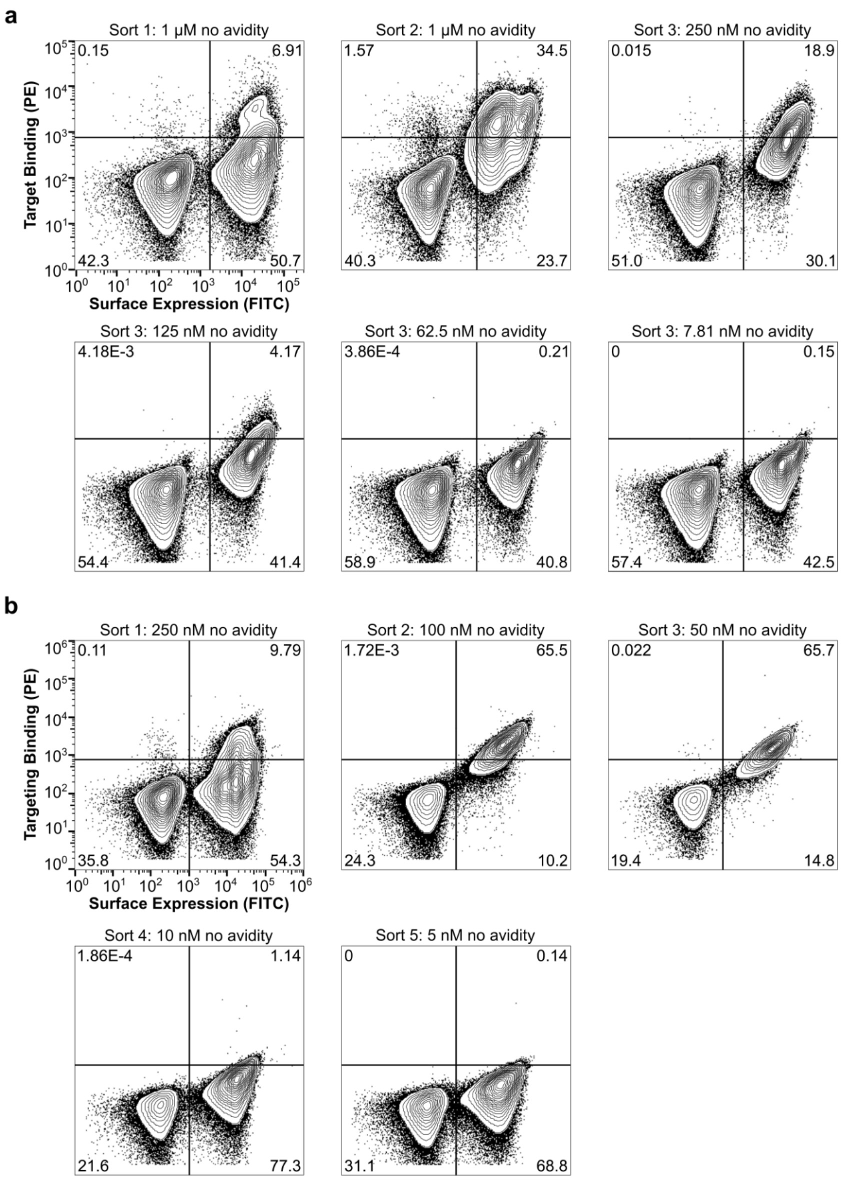

Supplementary Fig. 2: Affinity maturation of initial hits using yeast surface display.
a, Yeast displaying the SSM libraries for each design were pooled and incubated with fluorescently labeled hTLR3. Double positive cells were collected for sequencing and additional rounds of sorting for a total of 3 sorts. **b,** Yeast displaying the combinatorial libraries for each design were pooled and incubated with fluorescently labeled hTLR3. Double positive cells were collected for sequencing and additional rounds of sorting for a total of 5 sorts.

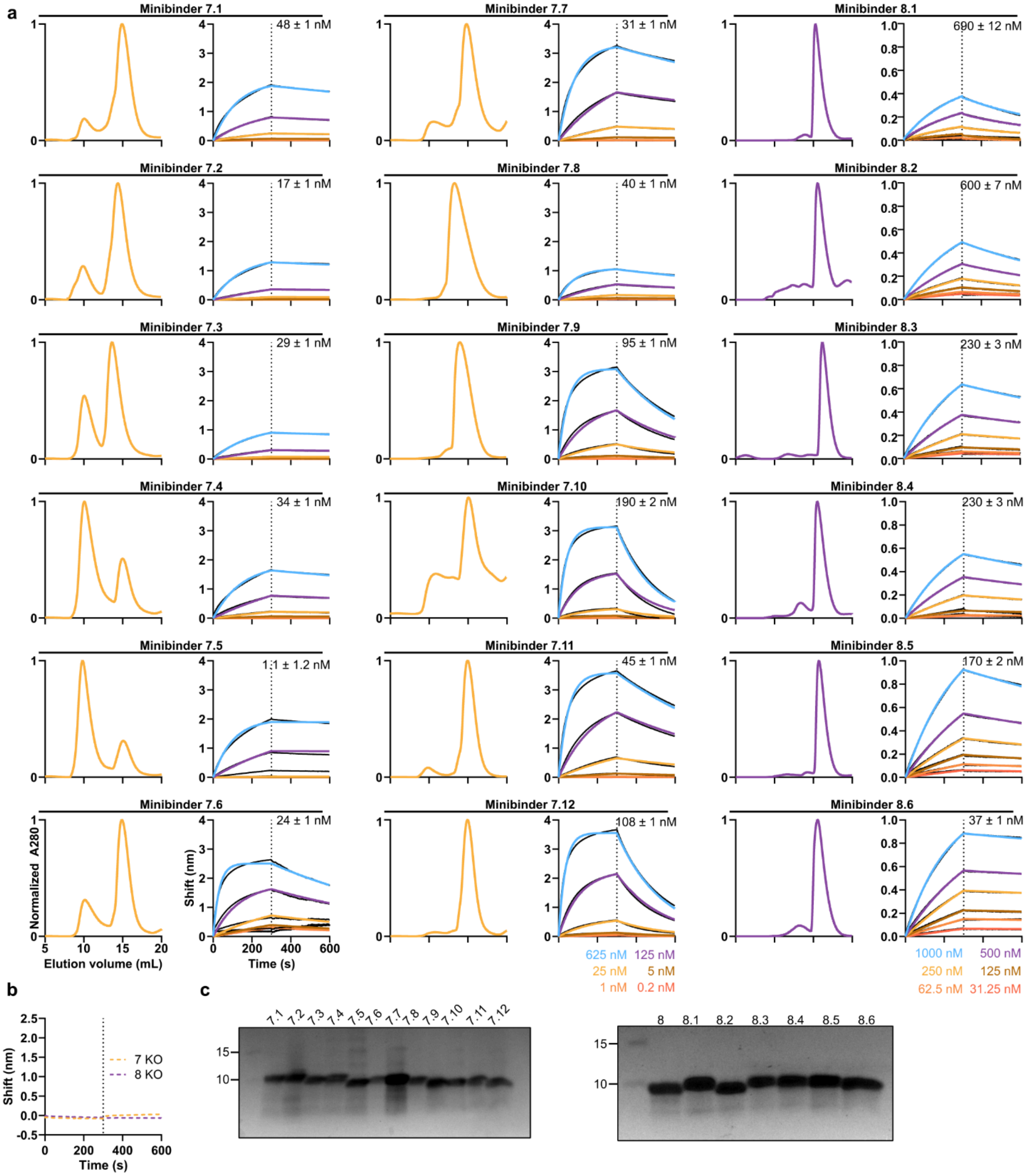

Supplementary Fig. 3: Biochemical and biophysical characterization of minibinder 7 and 8 variants.

a, SEC and BLI of affinity matured minibinders. KD values are listed for each minibinder. **b,** BLI of minibinders with interface knockout mutations. The KOs were tested for binding against 1 µM of hTLR3. **c,** Reducing SDS-PAGE of minibinders. The protein ladder (left) is labeled in kDa.

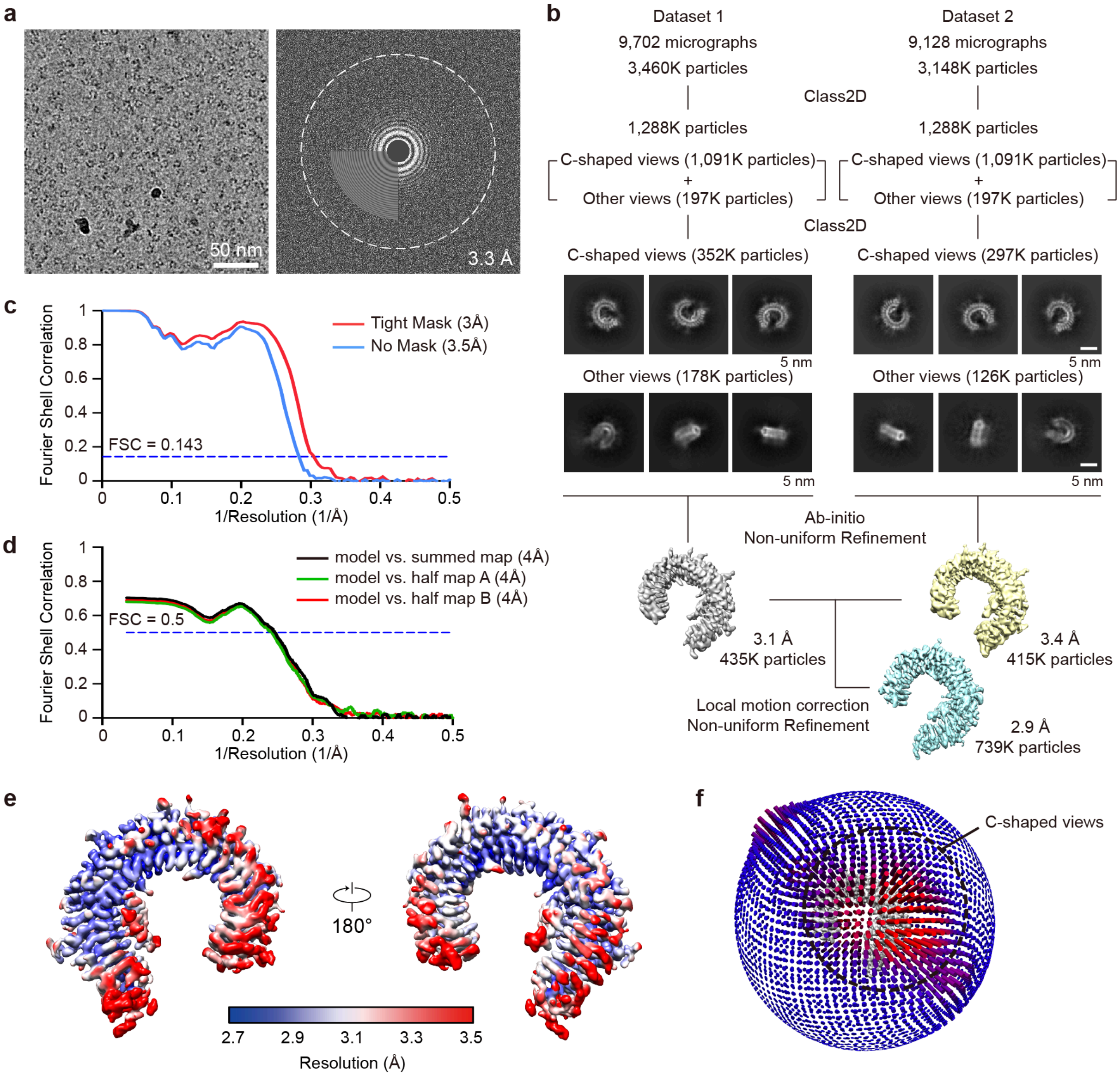

Supplementary Fig. 4: Cryo-EM analysis of TLR3/minibinder 7.7 complex.

a, Representative cryo-EM micrograph (left) and its Fourier transform (right). **b,** Data processing workflow of cryo-EM analysis and representative 2D class averages of the TLR3/minibinder 7.7 complex. **c,** Gold-standard Fourier shell correlation (FSC) between two independently refined half-maps in cryoSPARC (resolution cutoff at FSC = 0.143). **d,** FSC curves for cross-validation: model versus summed map (black), model versus half-map A (used in test refinement, green), and model versus half-map B (not used in test refinement, red). **e,** Final cryo-EM map colored by local resolution. **f,** Euler angle distribution of all particles used in the final 3D reconstructions. The height and color (from blue to red) of the cylinder bars are proportional to the number of particles in those views.

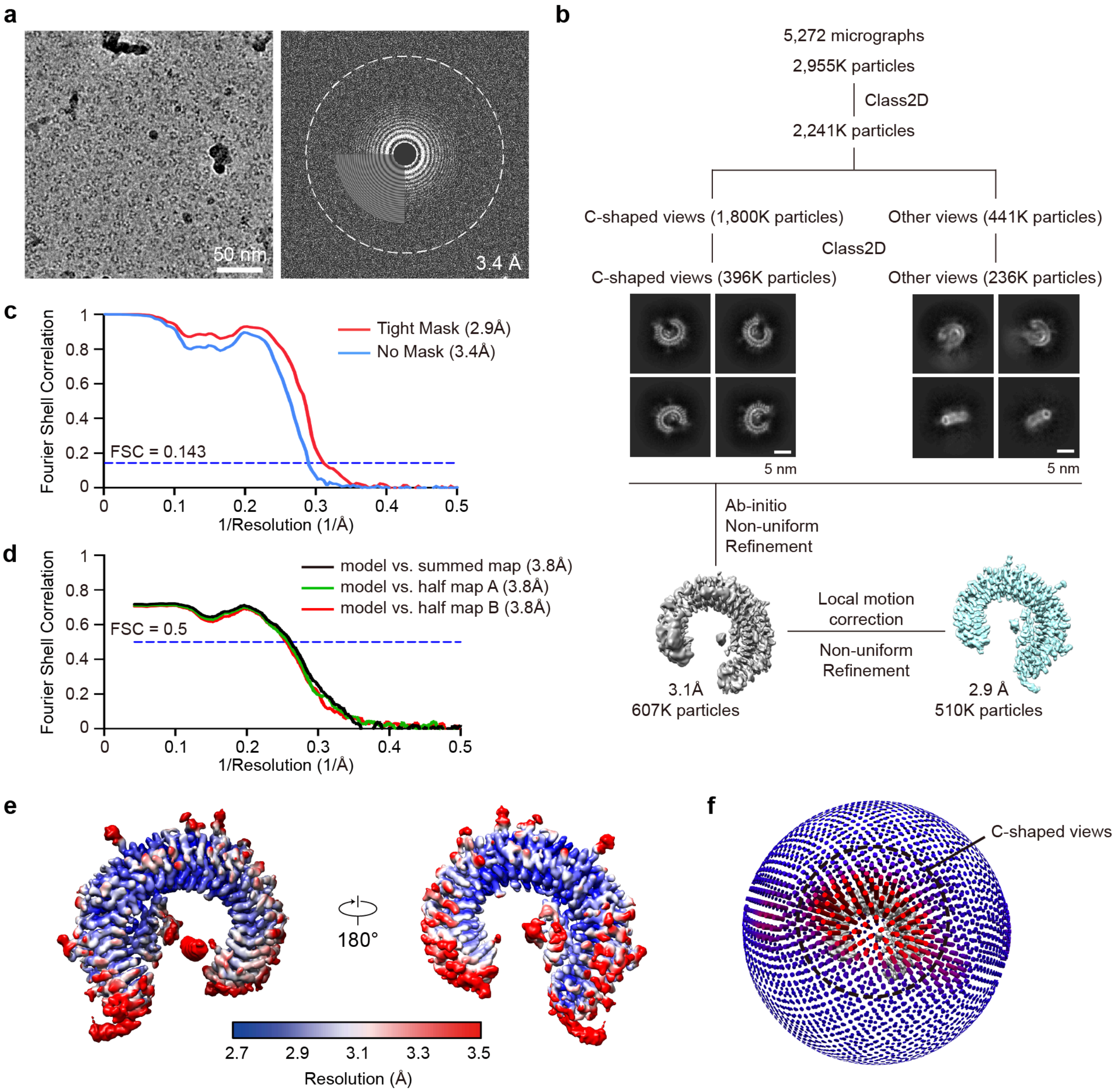

Supplementary Fig. 5: Cryo-EM analysis of TLR3/minibinder 8.6 complex.

a, Representative cryo-EM micrograph (left) and its Fourier transform (right). **b,** Data processing workflow of cryo-EM analysis and representative 2D class averages of the TLR3/minibinder 8.6 complex. **c,** Gold-standard Fourier shell correlation (FSC) between two independently refined half-maps in cryoSPARC (resolution cutoff at FSC = 0.143). **d,** FSC curves for cross-validation: model versus summed map (black), model versus half-map A (used in test refinement, green), and model versus half-map B (not used in test refinement, red). **e,** Final cryo-EM map colored by local resolution. **f,** Euler angle distribution of all particles used in the final 3D reconstructions. The height and color (from blue to red) of the cylinder bars are proportional to the number of particles in those views.

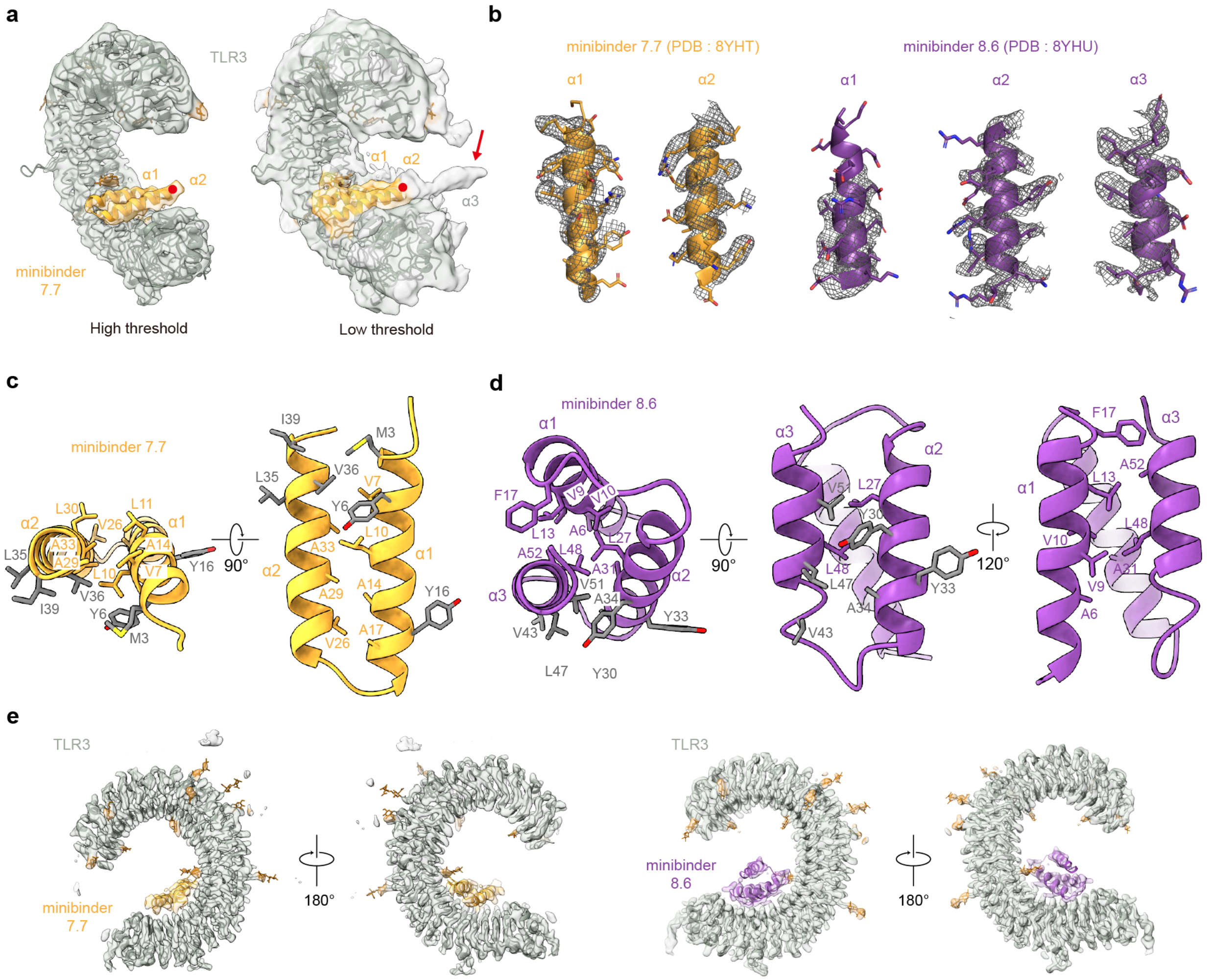

Supplementary Fig. 6: Cryo-EM map for TLR3/minibinder complex and hydrophobic residues in helices of minibinders.

a, Cryo-EM density for the third helix of minibinder 7.7 under high threshold (left, threshold = 0.2 σ) and low threshold (right, threshold = 0.08 σ). The C-terminal end of the second helix is marked with a red dot, and the potential density for the third helix is marked as α3 (red arrow). **b,** Cryo-EM map and model of minibinder 7.7 α1 (residues 1-20, contour level = 4), minibinder 7.7 α2 (residues 24-40, contour level = 5), minibinder 8.6 α1 (residues 1-16, contour level = 4), minibinder 8.6 α2 (residues 21-37, contour level = 3), minibinder 8.6 α3 (residues 42-56, contour level = 3). **c,d,** Hydrophobic residues in helices of minibinder 7.7 **(c)** and minibinder 8.6 **(d)**. Interacting residues in the hydrophobic core are shown as yellow (minibinder 7.7) and purple (minibinder 8.6) sticks. Residues involved in hydrophobic interactions between each minibinder and TLR3 are colored in gray. **e,** Cryo-EM map and model of TLR3/minibinder 7.7 (left, threshold = 0.09 σ) and TLR3/minibinder 8.6 (right, threshold = 0.1 σ) in two different views.

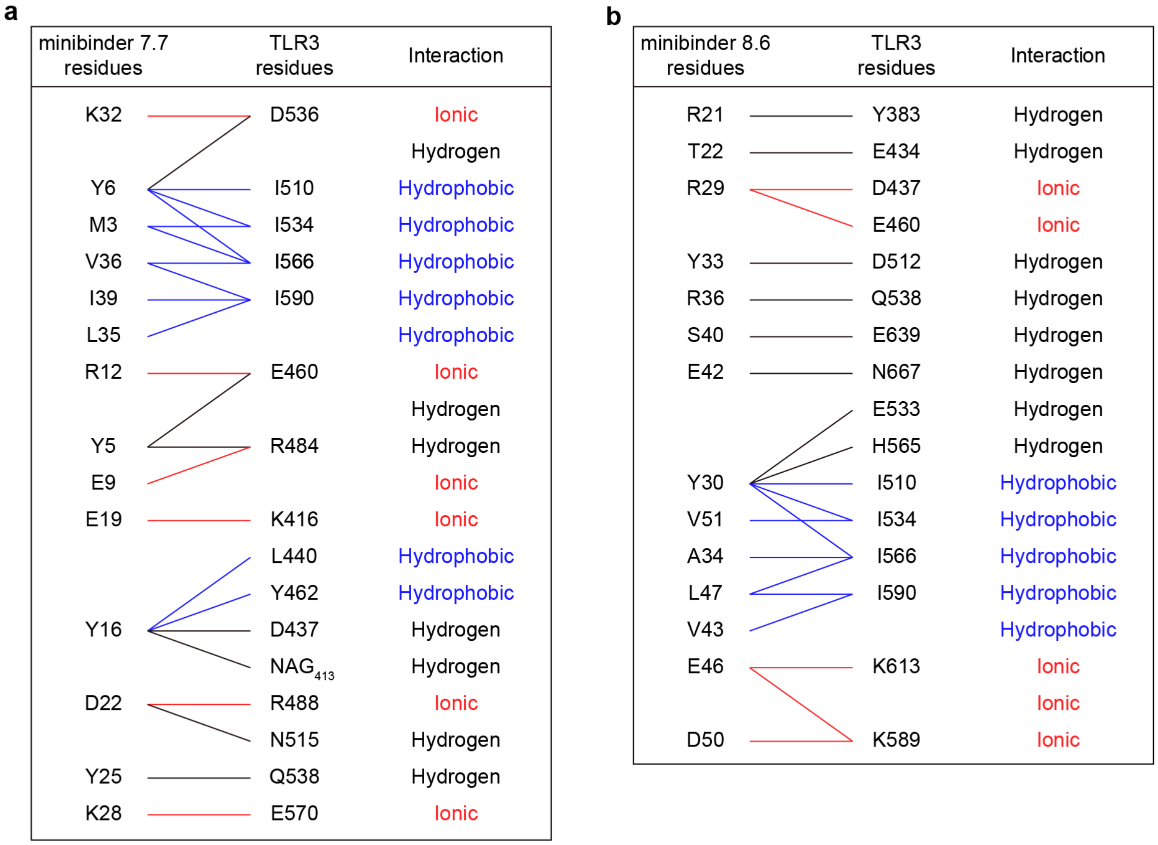

Supplementary Fig. 7: Interactions between minibinders and TLR3.
a,b, Residues making key interactions between TLR3 and minibinders 7.7 **(a)** and 8.6 **(b)** are provided, as well as the nature of each interaction.

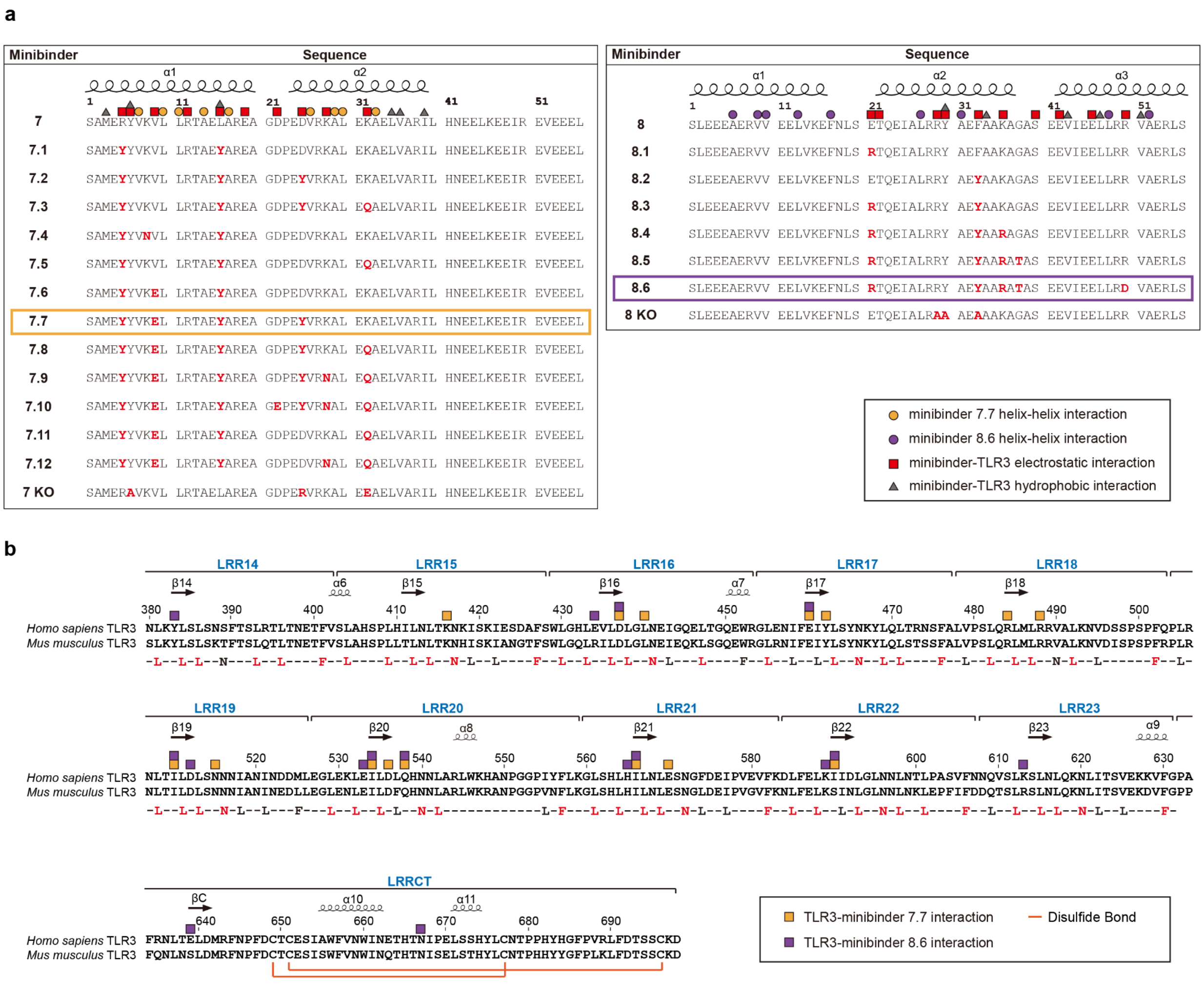

Supplementary Fig. 8: Sequence alignments of minibinder 7 and 8 derivatives and TLR3. **a,** Amino acid sequence alignments of left, minibinder 7, and 7.1–12, and right, 7 KO or 8, 8.1–6, and 8 KO. Residues mutated from the parental designs 7 or 8 are colored red. α-helices are noted above the alignment. Red squares and gray triangles indicate residues involved in electrostatic and hydrophobic interactions in the structures of minibinders 7.7 or 8.6 and TLR3, respectively. Yellow and purple circles indicate helix-helix interacting residues in minibinders 7.7 and 8.6, respectively. **b,** Amino acid sequence alignment of human (*H. sapiens*, UniProt: O15455) and mouse (*M. musculus*, Uniprot: Q99MB1) TLR3. The LRR consensus sequence of TLR3 (xLxxLxxLxLxxNxLxxLxxxxFx) is provided under the LRR motif alignment, and residues conserved in both sequences are colored red. Domains (LRRNT, LRR1–LRR23 motif, and LRRCT) and secondary structure (arrows for β stands and helices for α-helices) elements are noted above the alignment. Yellow and purple squares indicate TLR3 residues interacting with minibinders 7.7 and 8.6, respectively. Disulfide bonds are also indicated by orange lines. The sequence alignment was created using T-Coffee[1].

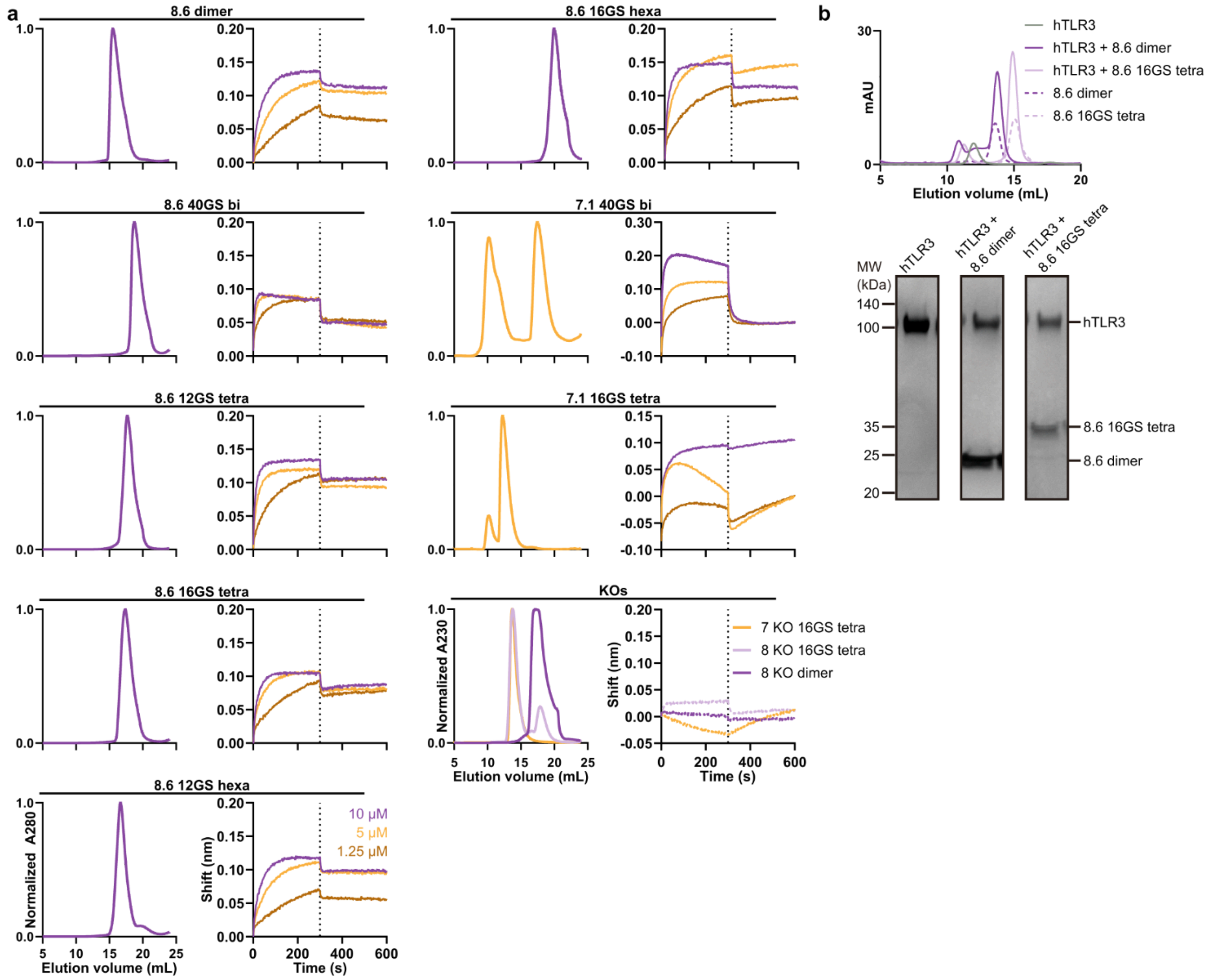

Supplementary Fig. 9: Characterization of multivalent constructs. a, SEC and BLI of multivalent constructs. Constructs were tested for binding at 10, 5, and 1.25 μM while interface knockout constructs were tested at 10 μM. b, Top, SEC of hTLR3 in complex with 8.6 16GS tetravalent or 8.6 dimer. Bottom, Reducing SDS-PAGE of peak fractions. The protein ladder (left) is labeled in kDa.

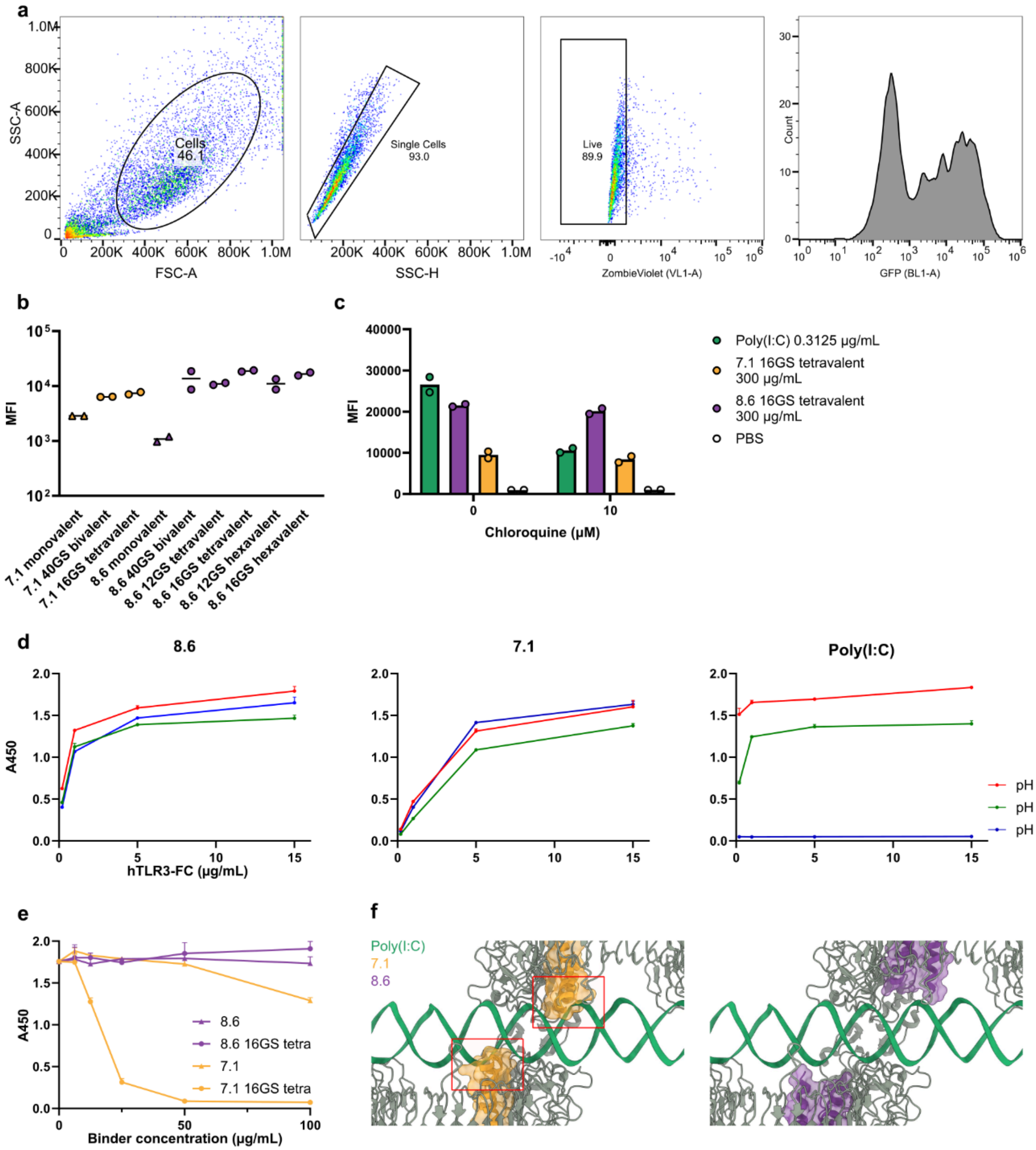

Supplementary Fig. 10: Activation of TLR3 by multivalent minibinders. a, Gating strategy for HEK293-TLR3hi assay. **b,** Various minibinder valencies and linker lengths were tested at 300 μg/mL each. **c,** Chloroquine, an endosome acidification inhibitor, decreased poly(I:C) signaling but not minibinder signaling. **d,** Capture ELISAs were performed using immobilized minibinder or immobilized poly(I:C). Dilutions of hTLR3-Fc were bound at pH 5.5, 6.5, and 7.4. **e,** Competition ELISAs were performed with immobilized poly(I:C). Monovalent or tetravalent binders were preincubated with hTLR3-Fc at pH 6.5. **f,** Cryo-EM models aligned to PDB 7wv5 reveal that minibinder 7.1 sterically clashes with bound Poly(I:C) (red boxes).

Supplementary Table 1. Amino acid sequences.

Construct*	Amino Acid Sequence
1	DR[**V**/I][**L**/Y]RA[**A**/R]AELAF[**K**/R]NLRID[**V/**I/L/M]EENDPEEVR[**Q/**S/T/Y]NLR[**H/**R]LET[**L/**Y]ARVLNDPEIERLVEEVKELLG
2	DEVWRILAQ[**M**/I][**T**/N][**H**/S]I[**L**/K/N/S/Q][**S**/N]HIDDPEE[**A**/N/S]YEV[**S**/D/N]RLF[**L**/Y]RVYELNDPEYARRALERAEEL
3	DN[**F**/Y]RYY[**V**/A/G]E[**Q**/N/S/Y]LLR[**S**/H/N/T]AEL[**L**/E/S]LEEGDPE[**E**/Y]AE[**L**/N/Q/R/S][**A**/R/S]L[**R**/H][**S**/N/Q]AKTVAR[**L**/I/V]ENDEELRRLVEELERRL
4	DD[**I**/N/T]FE[**F**/W]YIKYLIE[**A**/Q/N/T]AK[**R**/Y]AYEE[**G**/I]DDEAAENDLR[**T**/H]ARSAARVLGDEELRRLIEELERKI
5	DLEELIREARELLEKGNPYEAAK[**V**/Y]VLE[**A**/S]IHLAI[**Q**/M]K[**D**/R]DDE[**L**/R][**F**/I/L/M/S]LEAWRLYREILG
6	S[**A**/N/S/T]VELYLELLERS[**L**/M/T]R[**L**/F/Y]A[**V**/L]EAGDPEDAER[**I**/D][**L**/K]R[**K**/H/T]ARQIARVFNDPELEEIVERMEEILK
7	S[**A**/I/V]ME[**R**/H/Y]YVK[**V**/E]LLRTAE[**L/**Y]AREAG[**D**/E/H]PE[**D**/E/H/Y]VR[**K**/N/S/T][**A**/L]LE[**K**/F/Q]AE[L/M]VARILHNEELKEEIREVEEEL
8	SLEEEAERVVEELVKEFNLSET[**Q**/H]EI[**A**/H/Y]LRRY[**A**/E/H/R/Q]E[**F**/Y]AA[**K**/R]AGASEEVIEEL[**L**/V]RRVAERLS
9	DELERLAEEIVERLVKEYNLDFKQKLRLRIIAESLLEHGFDEELIELLLERDARRLS
10	DEELREVVERLVEKFQLSEEAKKVLEEVVKRLEERGFDEKLAKLRLYLVAARLSVEL
11	S[**D**/E]EA[**A**/Y]RIARE[**I**/V]LKLAEYAIKTDDPEALRLYNEAK[**N**/R]LL[**S**/H/N/R]EAEAKNSEEVLKKVEEVVRKAQKNVS
7.1	SAMEYYVKVLLRTAEYAREAGDPEDVRKALEKAELVARILHNEELKEEIREVEEEL
7.2	SAMEYYVKVLLRTAEYAREAGDPEYVRKALEKAELVARILHNEELKEEIREVEEEL
7.3	SAMEYYVKVLLRTAEYAREAGDPEYVRKALEQAELVARILHNEELKEEIREVEEEL
7.4	SAMEYYVNVLLRTAEYAREAGDPEDVRKALEKAELVARILHNEELKEEIREVEEEL
7.5	SAMEYYVKVLLRTAEYAREAGDPEDVRKALEQAELVARILHNEELKEEIREVEEEL
7.6	SAMEYYVKELLRTAEYAREAGDPEDVRKALEKAELVARILHNEELKEEIREVEEEL
7.7	SAMEYYVKELLRTAEYAREAGDPEYVRKALEKAELVARILHNEELKEEIREVEEEL
7.8	SAMEYYVKELLRTAEYAREAGDPEYVRKALEQAELVARILHNEELKEEIREVEEEL
7.9	SAMEYYVKELLRTAEYAREAGDPEYVRNALEQAELVARILHNEELKEEIREVEEEL

7.10	SAMEYYVKELLRTAEYAREAGEPEYVRNALEQAELVARILHNEELKEEIREVEEEL
7.11	SAMEYYVKELLRTAEYAREAGDPEDVRKALEQAELVARILHNEELKEEIREVEEEL
7.12	SAMEYYVKELLRTAEYAREAGDPEDVRNALEQAELVARILHNEELKEEIREVEEEL
7 KO	SAMERAVKVLLRTAELAREAGDPERVRKALEEAELVARILHNEELKEEIREVEEEL
8.1	SLEEEAERVVEELVKEFNLSRTQEIALRRYAEFAAKAGASEEVIEELLRRVAERLS
8.2	SLEEEAERVVEELVKEFNLSETQEIALRRYAEYAAKAGASEEVIEELLRRVAERLS
8.3	SLEEEAERVVEELVKEFNLSRTQEIALRRYAEYAAKAGASEEVIEELLRRVAERLS
8.4	SLEEEAERVVEELVKEFNLSRTQEIALRRYAEYAARAGASEEVIEELLRRVAERLS
8.5	SLEEEAERVVEELVKEFNLSRTQEIALRRYAEYAARATASEEVIEELLRRVAERLS
8.6	SLEEEAERVVEELVKEFNLSRTQEIALRRYAEYAARATASEEVIEELLRDVAERLS
8 KO	SLEEEAERVVEELVKEFNLSETQEIALRAAAEAAAKAGASEEVIEELLRRVAERLS
12GS tetravalent	**[BINDER]**GGSGGSGGSGGS**[BINDER]**GGSGGSGGSGGS**[BINDER]**GGSGGSGGSGGS**[BINDER]**
12GS hexavalent	**[BINDER]**GGSGGSGGSGGS**[BINDER]**GGSGGSGGSGGS**[BINDER]**GGSGGSGGSGGS**[BINDER]**GGSGGSGGSGGS**[BINDER]**GGSGGSGGSGGS**[BINDER]**
16GS tetravalent	**[BINDER]**GGSGGSGGSGGSGGSG**[BINDER]**GGSGGSGGSGGSGGSG**[BINDER]**GGSGGSGGSGGSGGSG**[BINDER]**
16GS hexavalent	**[BINDER]**GGSGGSGGSGGSGGSG**[BINDER]**GGSGGSGGSGGSGGSG**[BINDER]**GGSGGSGGSGGSGGSG**[BINDER]**GGSGGSGGSGGSGGSG**[BINDER]**GGSGGSGGSGGSGGSG**[BINDER]**
40GS bivalent	**[BINDER]**GGSGGSGGSEGGSGGGSEGSGGSEGGSGGSGEGSGGSGGS**[BINDER]**
dimer	GSHENKQVEEILRLEKEIEDLQRMKERQELSLTEASLQKLQLEDKVEELLSKNYHLENEVARLKKLVGEGGSGGSGGSGGSGGSGGSGGSGGS[**BINDER]**
TLR3 ectodomain	MLLVNQSHQGFNKEHTSKMVSAIVLYVLLAAAAHSAFAKCTVSHEVADCSHLKLTQVPDDLPTNITVLNLTHNQLRRLPAANFTRYSQLTSLDVGFNTISKLEPELCQKLPMLKVLNLQHNELSQLSDKTFAFCTNLTELHLMSNSIQKIKNNPFVKQKNLITLDLSHNGLSSTKLGTQVQLENLQELLLSNNKIQALKSEELDIFANSSLKKLELSSNQIKEFSPGCFHAIGRLFGLFLNNVQLGPSLTEKLCLELANTSIRNLSLSNSQLSTTSNTTFLGLKWTNLTMLDLSYNNLNVVGNDSFAWLPQLEYFFLEYNNIQHLFSHSLHGLFNVRYLNLKRSFTKQSISLASLPKIDDFSFQWLKCLEHLNMEDNDIPGIKSNMFTGLINLKYLSLSNSFTSLRTLTNETFVSLAHSPLHILNLTKNKISKIESDAFSWLGHLEVLDLGLNEIGQELTGQEWRGLENIFEIYLSYNKYLQLTRNSFALVPSLQRLMLRRVALKNVDSSPSPFQPLRNLTILDLSNNNIANINDDMLEG

LEKLEILDLQHNNLARLWKHANPGGPIYFLKGLSHLHILNLESNGFDEIPVEVFKDLFELK
IIDLGLNNLNTLPASVFNNQVSLKSLNLQKNLITSVEKKVFGPAFRNLTELDMRFNPFDC
TCESIAWFVNWINETHTNIPELSSHYLCNTPPHYHGFPVRLFDTSSCKDSAGGSHHHH
HHGGSGLNDIFEAQKIEWHE

---

* For minibinders 1-11, the amino acids encoded by degenerate codons in the combinatorial libraries are listed in brackets, with the original amino acid bolded.

Supplementary Table 2. Association and dissociation rates for TLR3/minibinder interactions, determined by BLI

Minibinder	K_{on} ($M^{-1}s^{-1}$)	K_{off} (s^{-1})
1	N.D.*	N.D.
2	N.D.	N.D.
3	1.1E+04	4.5E-03
4	6.9E+03	1.7E-03
5	N.D.	N.D.
6	1.1E+04	4.9E-04
7	4.8E+03	2.1E-03
8	2.5E+03	3.0E-03
9	4.1E+03	6.3E-03
10	N.D.	N.D.
11	N.D.	N.D.
7.1	7.9E+03	3.4E-04
7.2	9.3E+03	1.6E-04
7.3	8.0E+03	2.3E-04
7.4	1.0E+04	3.6E-04
7.5	1.6E+04	1.8E-05
7.6	5.0E+04	1.2E-03
7.7	1.9E+04	5.9E-04
7.8	2.0E+04	7.7E-04
7.9	2.9E+04	2.7E-03
7.10	3.0E+04	5.7E-03
7.11	3.0E+04	1.4E-03
7.12	4.0E+04	4.4E-03
8.1	2.8E+03	1.9E-03
8.2	2.1E+03	1.3E-03
8.3	2.9E+03	6.5E-04
8.4	2.9E+03	6.5E-04
8.5	3.3E+03	5.6E-04
8.6	4.5E+03	1.7E-04

*N.D. indicates no binding data.

Supplementary Table 3. Cryo-EM data collection, refinement, and validation statistics

	TLR3/minibinder 7.7	TLR3/minibinder 8.6
**Data collection and processing**		
Magnification	130,000	130,000
Voltage (kV)	300	300
Electron exposure ($e^-/Å^2$)	68.9	69.8
Defocus range (μm)	-0.8 – -2.2	-0.8 – -2.0
Pixel size (Å)	0.664	0.664
Symmetry imposed	C1	C1
Initial particle images (no.)	6,608,485	2,955,749
Final particle images (no.)	739,755	510,475
Map resolution (Å)	2.88	2.88
FSC threshold	0.143	0.143
**Refinement**		
Initial model used	AF2 prediction	AF2 prediction
Map resolution (Å)	2.88	2.88
FSC threshold	0.143	0.143
Map sharpening B factor ($Å^2$)	0	0
**Model composition**		
Non Hydrogen atoms	5,845	5,990
Protein residues	703	719
Ligands	14	15
**B factors ($Å^2$)**		
Protein	75.39	64.40
Ligand	77.43	70.91
**R.m.s. deviations**		
Bond lengths (Å)	0.26	0.26
Bond angles (°)	0.51	0.51
**Validation**		
MolProbity score	2.33	2.15
Clashscore	11	11
Poor rotamers (%)	0	0
**Ramachandran plot**		
Favored (%)	88	87
Allowed (%)	12	13
Outliers (%)	0	0

References

1. Poirot, O., O'Toole, E. & Notredame, C. Tcoffee@igs: A web server for computing, evaluating and combining multiple sequence alignments. *Nucleic Acids Research* **31**, 3503-3506 (2003).
