# Supplementary material for: De novo design of protein minibinder agonists of TLR3
Source: Nat Commun. 2025 Jan 31;16:1234. doi: 10.1038/s41467-025-56369-w (PMC11785957; doi:10.1038/s41467-025-56369-w)
Supplement: Supplementary file 2 — Reporting Summary [file 41467_2025_56369_MOESM2_ESM.pdf]

nature portfolio

Corresponding author(s): Ho Min Kim, Neil P. King

Last updated by author(s): Jan 13, 2025

Reporting Summary

Nature Portfolio wishes to improve the reproducibility of the work that we publish. This form provides structure for consistency and transparency in reporting. For further information on Nature Portfolio policies, see our Editorial Policies and the Editorial Policy Checklist.

Statistics

For all statistical analyses, confirm that the following items are present in the figure legend, table legend, main text, or Methods section.

n/a	Confirmed	
☐	☒	The exact sample size (*n*) for each experimental group/condition, given as a discrete number and unit of measurement
☒	☐	A statement on whether measurements were taken from distinct samples or whether the same sample was measured repeatedly
☒	☐	The statistical test(s) used AND whether they are one- or two-sided<br>*Only common tests should be described solely by name; describe more complex techniques in the Methods section.*
☒	☐	A description of all covariates tested
☒	☐	A description of any assumptions or corrections, such as tests of normality and adjustment for multiple comparisons
☐	☒	A full description of the statistical parameters including central tendency (e.g. means) or other basic estimates (e.g. regression coefficient) AND variation (e.g. standard deviation) or associated estimates of uncertainty (e.g. confidence intervals)
☒	☐	For null hypothesis testing, the test statistic (e.g. *F*, *t*, *r*) with confidence intervals, effect sizes, degrees of freedom and *P* value noted<br>*Give P values as exact values whenever suitable.*
☒	☐	For Bayesian analysis, information on the choice of priors and Markov chain Monte Carlo settings
☒	☐	For hierarchical and complex designs, identification of the appropriate level for tests and full reporting of outcomes
☒	☐	Estimates of effect sizes (e.g. Cohen's *d*, Pearson's *r*), indicating how they were calculated

Our web collection on statistics for biologists contains articles on many of the points above.

Software and code

Policy information about availability of computer code

Data collection	Computational design was performed using the Rosetta software suite. More info can be found at https://github.com/rifdock/rifdock. Yeast flow cytometry data was collected on Sony Cell Sorter Software. Unicorn 7.3 was used to collect SEC traces. BLI data was collected on Octet Data Acquisition software v12.0.2.11. CD spectra were collected in Spectra Manager. Cryo-EM data was collected automatically using Thermo Scientific EPU v2.9 at Institute for Basic Science (IBS, Republic of Korea). AlphaFold2 was used to predict structures. Data collection statistics are shown in Supplementary Table 3. Mammalian flow cytometry data was collected on Attune Cytometric Software v6.1.0. ELISA data was collected using an EPOCH2 plate reader.
Data analysis	BLI data was analyzed using ForteBio Data Analysis Software v9.0.0.14. Flow cytometry data was analyzed using FlowJo 10.9.0. BLI data, mean fluorescence intensity values, CD data, and SEC traces were graphed using Graphpad Prism v10.1.0. CryoSPARC v4.2.1, MotionCor2, CTFFIND4 v4.1.10, COOT v0.8.9.2 and PHENIX v1.19.2 were used for data processing, model building and structure refinement. All structure figures were generated using PyMOL v2.3.1 and UCSF ChimeraX v1.3.

For manuscripts utilizing custom algorithms or software that are central to the research but not yet described in published literature, software must be made available to editors and reviewers. We strongly encourage code deposition in a community repository (e.g. GitHub). See the Nature Portfolio guidelines for submitting code & software for further information.

Data

Policy information about availability of data

All manuscripts must include a data availability statement. This statement should provide the following information, where applicable:

- Accession codes, unique identifiers, or web links for publicly available datasets
- A description of any restrictions on data availability
- For clinical datasets or third party data, please ensure that the statement adheres to our policy

The cryo-EM density maps of TLR3/minibinder 7.7 and TLR3/minibinder 8.6 complex have been deposited in the Electron Microscopy Data Bank (EMDB; https://www.ebi.ac.uk/pdbe/emdb/) under accession number EMD-39300 and EMD-39301, respectively. The atomic coordinates of TLR3/minibinder 7.7 and TLR3/minibinder 8.6 complex have been deposited in the Protein Data Bank (PDB; https://www.rcsb.org) with accession code 8YHT and 8YHU, respectively. The structural data from PDB used in this study are listed below: 7WV5 [https://doi.org/10.2210/pdb7WV5/pdb] (TLR3 ectodomain/poly(I:C) complex), 1ZIW [https://doi.org/10.2210/pdb1ZIW/pdb] (hTLR3 ectodomain), 7WV3 [https://doi.org/10.2210/pdb7WV3/pdb] (TLR3 linear cluster), 7C76 [https://doi.org/10.2210/pdb7C76/pdb] (hTLR3/UNC93B1 complex), 2N9B [https://doi.org/10.2210/pdb2N9B/pdb] (antiparallel dimer). All other data are available in the manuscript or the supplementary materials. All other data are available in the manuscript or the supplementary materials. Further information and requests for resources and reagents should be directed to and will be fulfilled by the corresponding authors (hm_kim@kaist.ac.kr) (neilking@uw.edu).

Research involving human participants, their data, or biological material

Policy information about studies with human participants or human data. See also policy information about sex, gender (identity/presentation), and sexual orientation and race, ethnicity and racism.

Reporting on sex and gender	*Use the terms sex (biological attribute) and gender (shaped by social and cultural circumstances) carefully in order to avoid confusing both terms. Indicate if findings apply to only one sex or gender; describe whether sex and gender were considered in study design; whether sex and/or gender was determined based on self-reporting or assigned and methods used. Provide in the source data disaggregated sex and gender data, where this information has been collected, and if consent has been obtained for sharing of individual-level data; provide overall numbers in this Reporting Summary. Please state if this information has not been collected. Report sex- and gender-based analyses where performed, justify reasons for lack of sex- and gender-based analysis.*
Reporting on race, ethnicity, or other socially relevant groupings	*Please specify the socially constructed or socially relevant categorization variable(s) used in your manuscript and explain why they were used. Please note that such variables should not be used as proxies for other socially constructed/relevant variables (for example, race or ethnicity should not be used as a proxy for socioeconomic status). Provide clear definitions of the relevant terms used, how they were provided (by the participants/respondents, the researchers, or third parties), and the method(s) used to classify people into the different categories (e.g. self-report, census or administrative data, social media data, etc.) Please provide details about how you controlled for confounding variables in your analyses.*
Population characteristics	*Describe the covariate-relevant population characteristics of the human research participants (e.g. age, genotypic information, past and current diagnosis and treatment categories). If you filled out the behavioural & social sciences study design questions and have nothing to add here, write "See above."*
Recruitment	*Describe how participants were recruited. Outline any potential self-selection bias or other biases that may be present and how these are likely to impact results.*
Ethics oversight	*Identify the organization(s) that approved the study protocol.*

Note that full information on the approval of the study protocol must also be provided in the manuscript.

Field-specific reporting

Please select the one below that is the best fit for your research. If you are not sure, read the appropriate sections before making your selection.

☒ Life sciences ☐ Behavioural & social sciences ☐ Ecological, evolutionary & environmental sciences

For a reference copy of the document with all sections, see nature.com/documents/nr-reporting-summary-flat.pdf

Life sciences study design

All studies must disclose on these points even when the disclosure is negative.

Sample size	The number of designs was ordered based off of previous success rates for this design pipeline. For cryo-EM samples, twelve grids of the TLR3/minibinder complex were pre-screened to identify the optimal grid for data collection. Sufficient cryo-EM raw movies were collected until cryo-EM map with adequate resolution for model building can be achieved.
Data exclusions	For Cryo-EM data, all acquired images were analyzed and parts were later excluded as is usual for such image processing studies. Misaligned image segments were excluded from averages based on cross-correlation scores and visual analysis (described in Methods section and Supplementary Figure 5 and 6).

Replication	For the cell signaling assay, constructs were tested in duplicate and data was reproducible. ELISA data was tested in duplicate and data was reproducible. BLI data was repeated at least twice and data was reproducible. Yeast display was not repeated.
Randomization	For Cryo-EM data collection, meshes on the cryo-EM grids were randomly selected. For cryo-EM data processing, randomization was performed based on standard algorithm in cryoSPARC v4.2.1. For other experiments and analyses, it was unnecessary to perform randomized experiments in the current study.
Blinding	No blinding was performed as a single scientist carried out the experiments.

Reporting for specific materials, systems and methods

We require information from authors about some types of materials, experimental systems and methods used in many studies. Here, indicate whether each material, system or method listed is relevant to your study. If you are not sure if a list item applies to your research, read the appropriate section before selecting a response.

Materials & experimental systems

n/a	Involved in the study
☐	☒ Antibodies
☐	☒ Eukaryotic cell lines
☒	☐ Palaeontology and archaeology
☒	☐ Animals and other organisms
☒	☐ Clinical data
☒	☐ Dual use research of concern
☒	☐ Plants

Methods

n/a	Involved in the study
☒	☐ ChIP-seq
☐	☒ Flow cytometry
☒	☐ MRI-based neuroimaging

Antibodies

Antibodies used	anti-c-myc-FITC conjugated (ICL) (Cat# CMYC-45F), anti-human IgG FC HRP (abcam) (Cat# ab97225)
Validation	Verified by manufacturer

Eukaryotic cell lines

Policy information about cell lines and Sex and Gender in Research

Cell line source(s)	1. Expi293F cells: Thermo Fisher, Cat# A14527. Expi293F cells were derived from HEK293 cells that is derived from a human fetus. 2. HEK293-TLR3hi (Dr. Joshua Leonard). HEK293-TLR3hi is a HEK293 cell line that is derived from a human fetus. The cell line was modified to express hTLR3 on the cell surface.
Authentication	The cell lines were not authenticated.
Mycoplasma contamination	The purchased cell lines were not tested for mycoplasma contamination, because we immediately used after purchase from manufacturer. The cell line from collaborator was tested by the collaborator.
Commonly misidentified lines (See ICLAC register)	No commonly misidentified cell lines were used in this study.

Plants

Seed stocks	*Report on the source of all seed stocks or other plant material used. If applicable, state the seed stock centre and catalogue number. If plant specimens were collected from the field, describe the collection location, date and sampling procedures.*
Novel plant genotypes	*Describe the methods by which all novel plant genotypes were produced. This includes those generated by transgenic approaches, gene editing, chemical/radiation-based mutagenesis and hybridization. For transgenic lines, describe the transformation method, the number of independent lines analyzed and the generation upon which experiments were performed. For gene-edited lines, describe the editor used, the endogenous sequence targeted for editing, the targeting guide RNA sequence (if applicable) and how the editor was applied.*
Authentication	*Describe any authentication procedures for each seed stock used or novel genotype generated. Describe any experiments used to assess the effect of a mutation and, where applicable, how potential secondary effects (e.g. second site T-DNA insertions, mosiacism, off-target gene editing) were examined.*

Flow Cytometry

Plots

Confirm that:

- [x] The axis labels state the marker and fluorochrome used (e.g. CD4-FITC).
- [x] The axis scales are clearly visible. Include numbers along axes only for bottom left plot of group (a 'group' is an analysis of identical markers).
- [x] All plots are contour plots with outliers or pseudocolor plots.
- [x] A numerical value for number of cells or percentage (with statistics) is provided.

Methodology

Sample preparation	Yeast display: Yeast cells are incubated with target protein, anti-myc antibody conjugated with FITC, and streptavidin conjugated with PE (ThermoFisher, #S866). The cells were washed with FACS buffer. See methods for experimental details.<br><br>Mammalian flow: Cells were dissociated with warm PBS. Zombie Violet Viability stain was added (BioLegend). The cells were washed with FACS buffer. See methods for experimental details.
Instrument	Yeast: Sony SH800 Cell Sorter. Mammalian: Attune CytPix Flow Cytometer.
Software	FlowJo10.9.0
Cell population abundance	*Describe the abundance of the relevant cell populations within post-sort fractions, providing details on the purity of the samples and how it was determined.*
Gating strategy	Yeast display: Single cells were selected. Cells labeled without the target protein were used as a negative control and all of the cells showing binding signal were collected.<br><br>Mammalian flow: Single, live cells were selected for. PBS alone was used as a negative control. Histograms showing GFP+ cells are shown in Fig.4. Gating strategy shown Extended Data. Fig. 9.

- [x] Tick this box to confirm that a figure exemplifying the gating strategy is provided in the Supplementary Information.
