# Supplementary material for: De novo design of protein minibinder agonists of TLR3
Source: Nat Commun. 2025 Jan 31;16:1234. doi: 10.1038/s41467-025-56369-w (PMC11785957; doi:10.1038/s41467-025-56369-w)

Minibinder 1

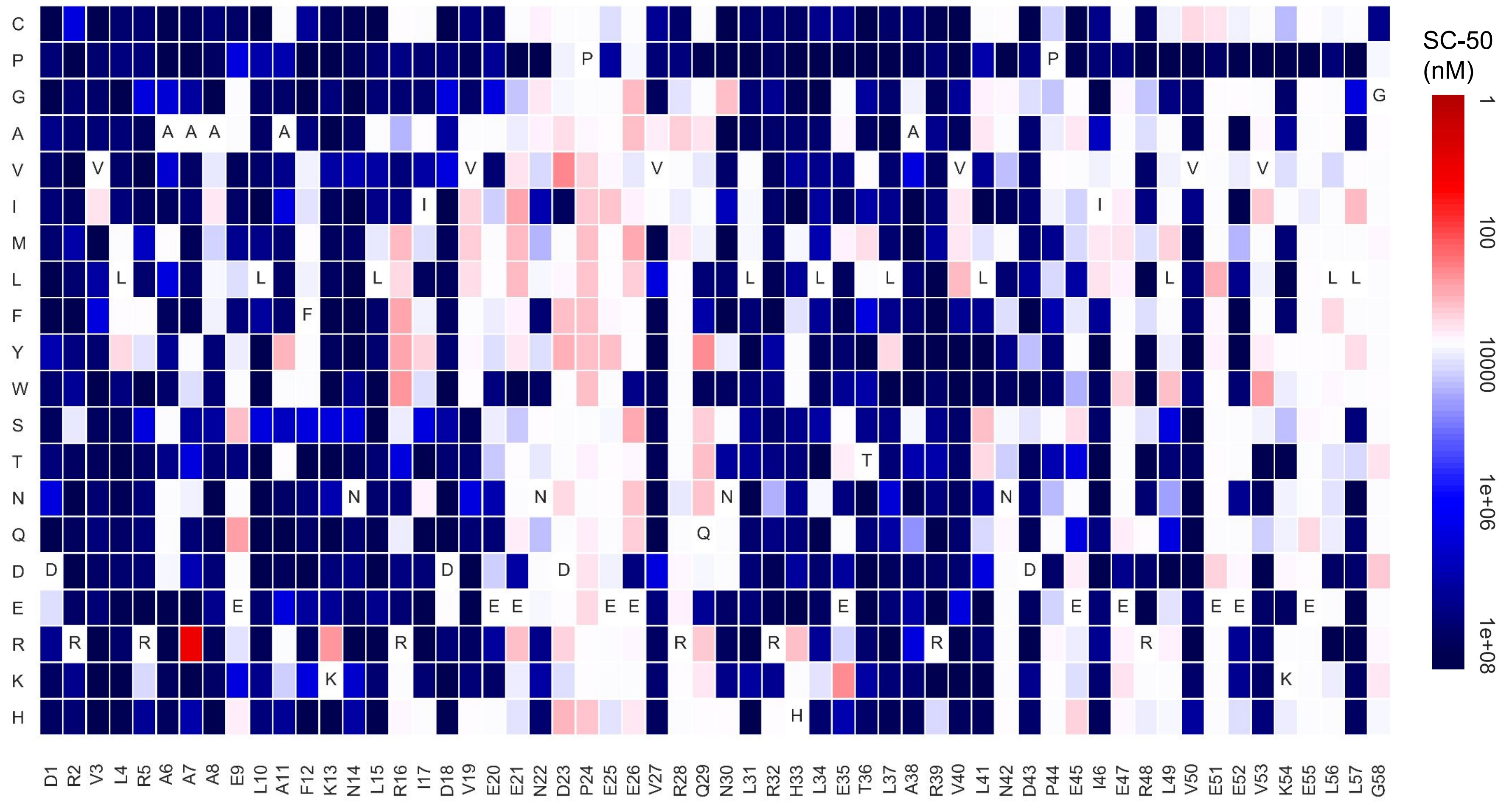

Minibinder 2

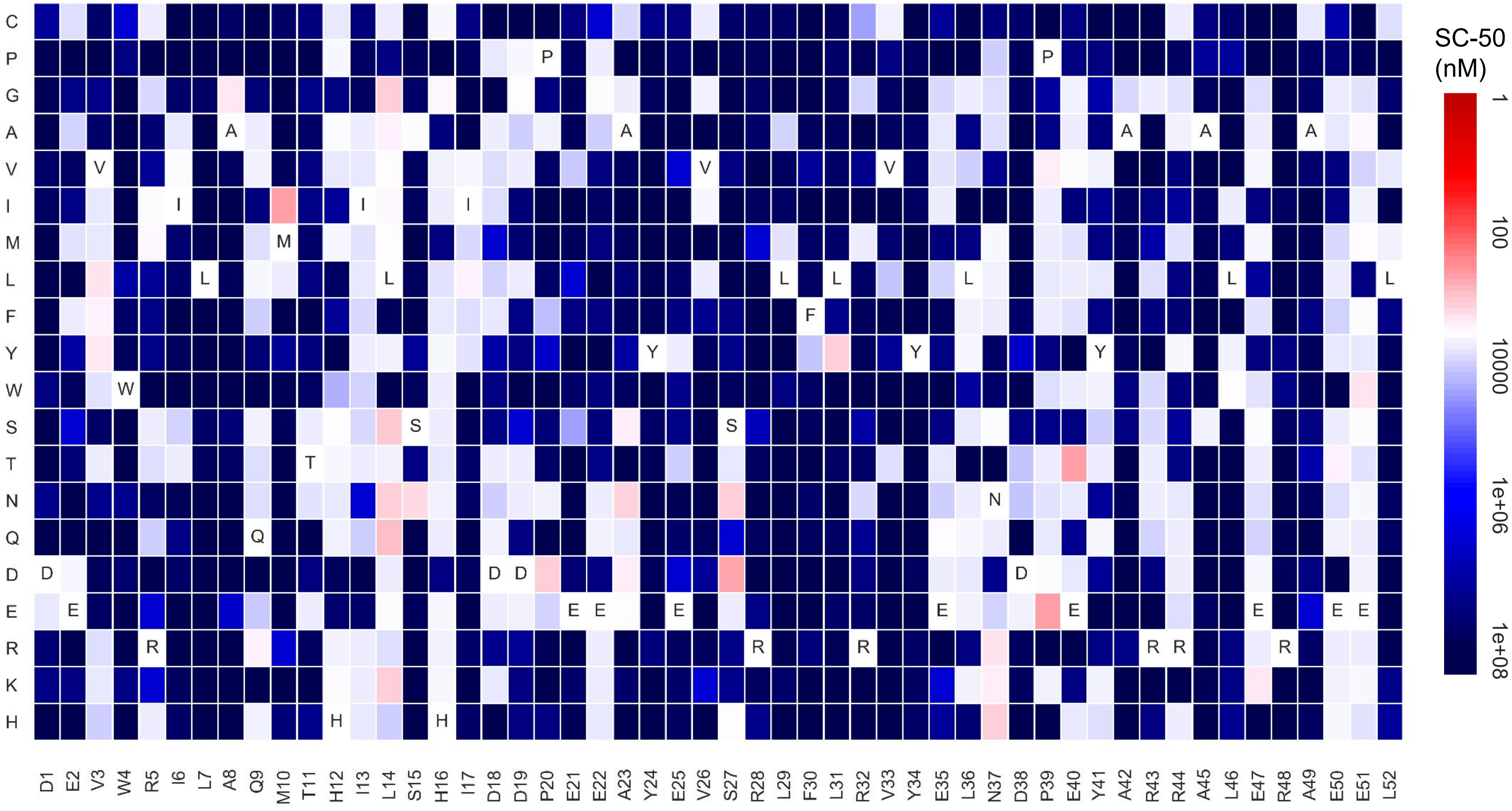

Minibinder 3

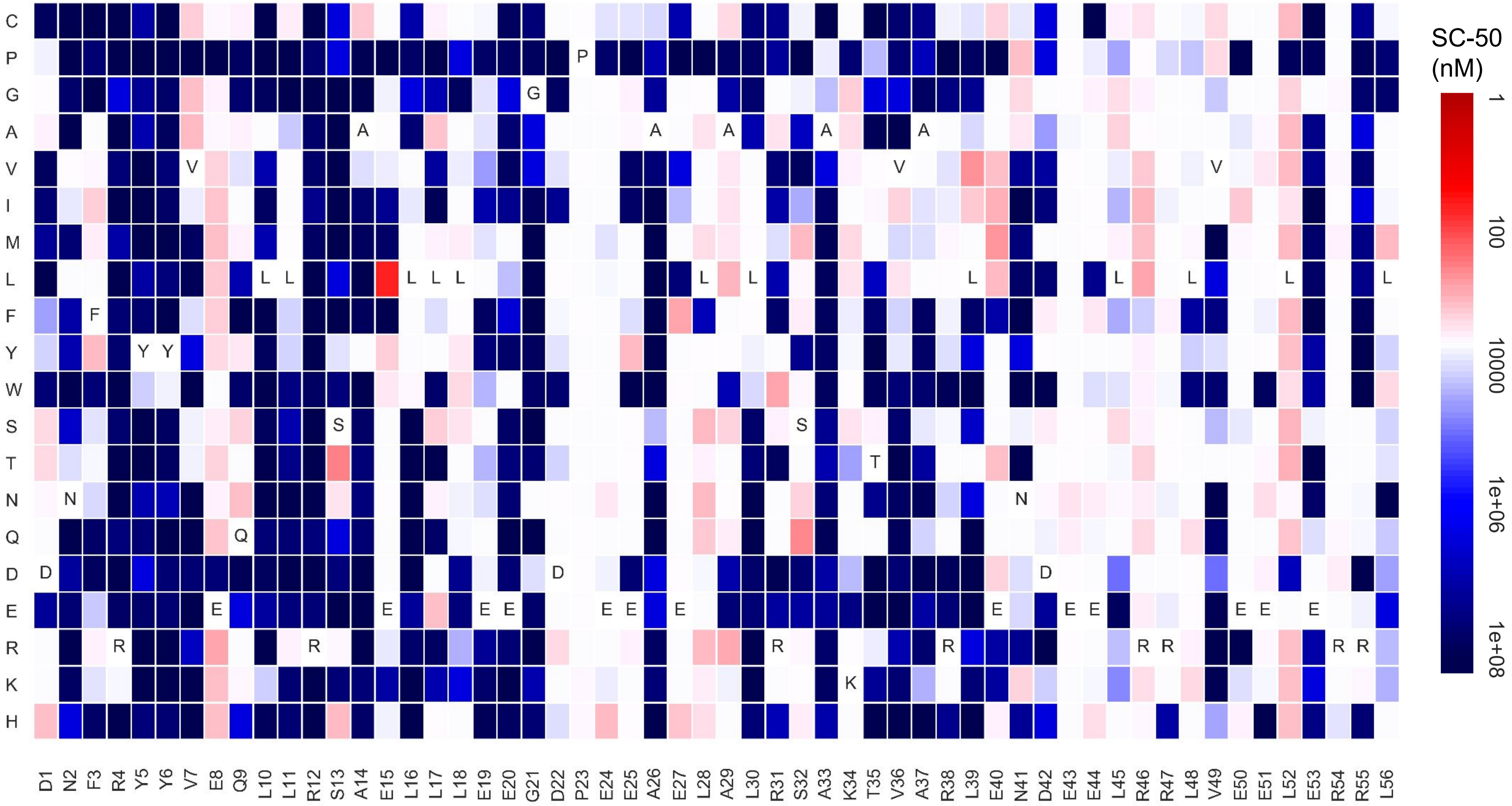

Minibinder 4

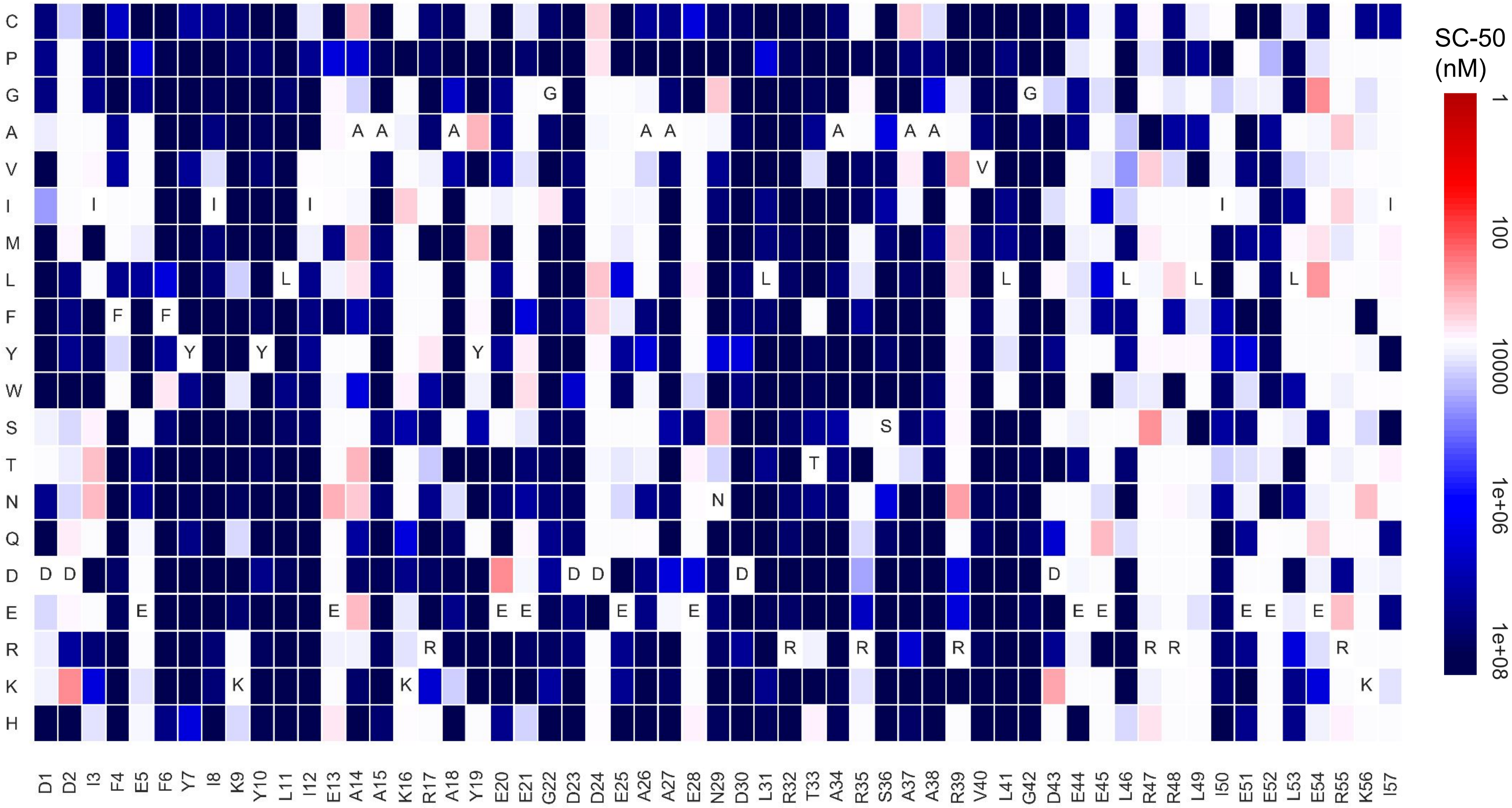

Minibinder 5

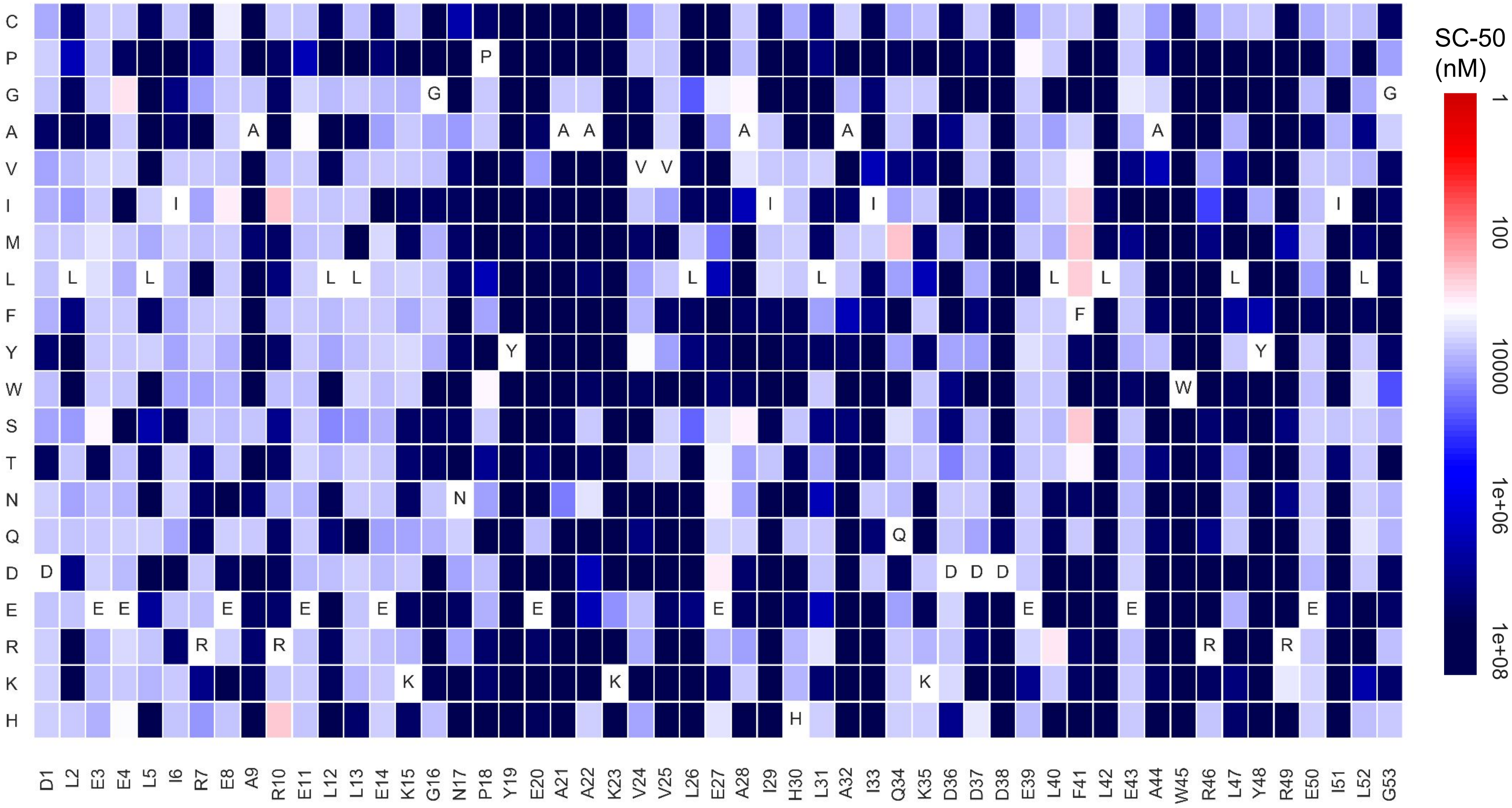

Minibinder 6

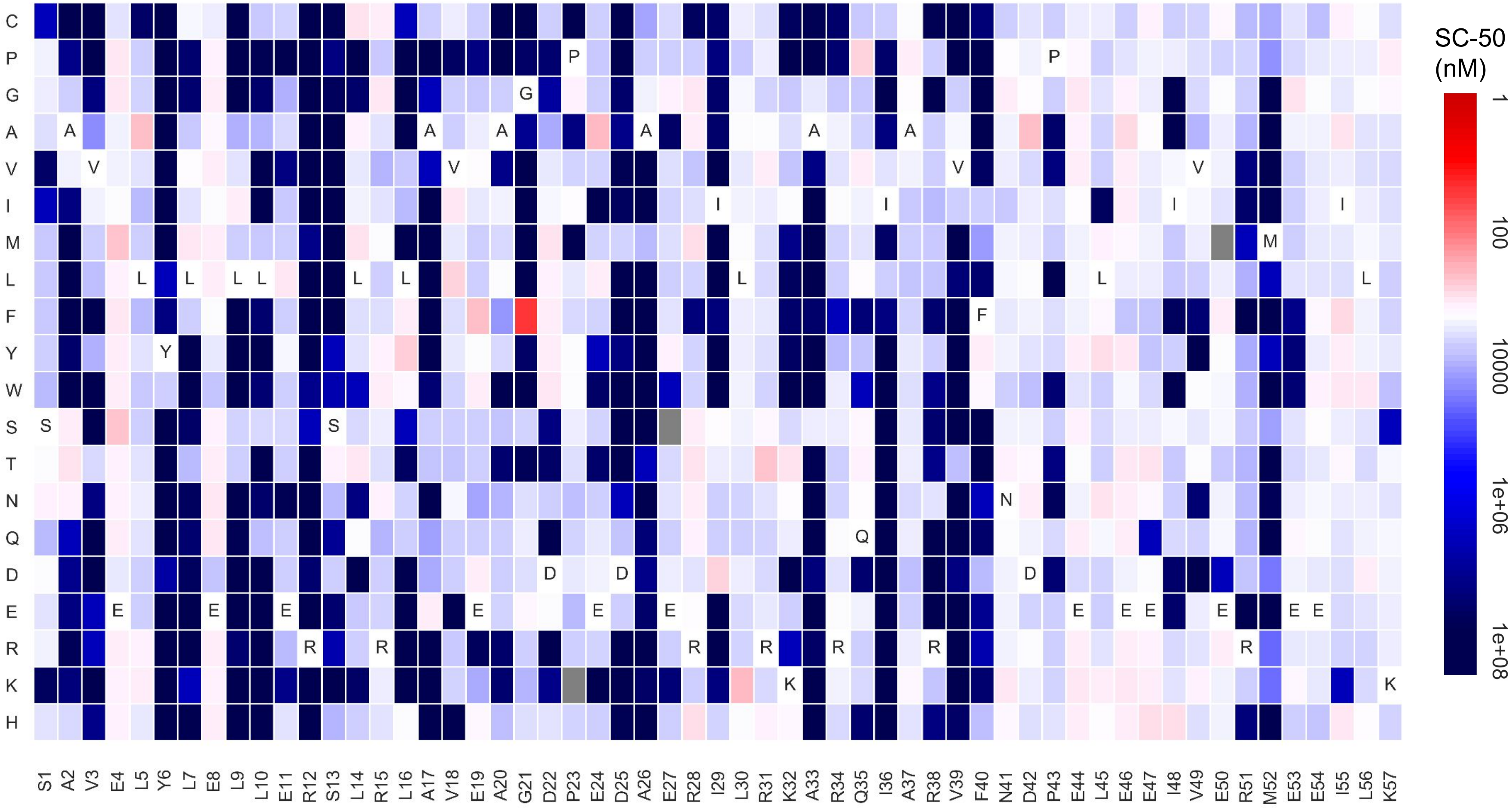

Minibinder 7

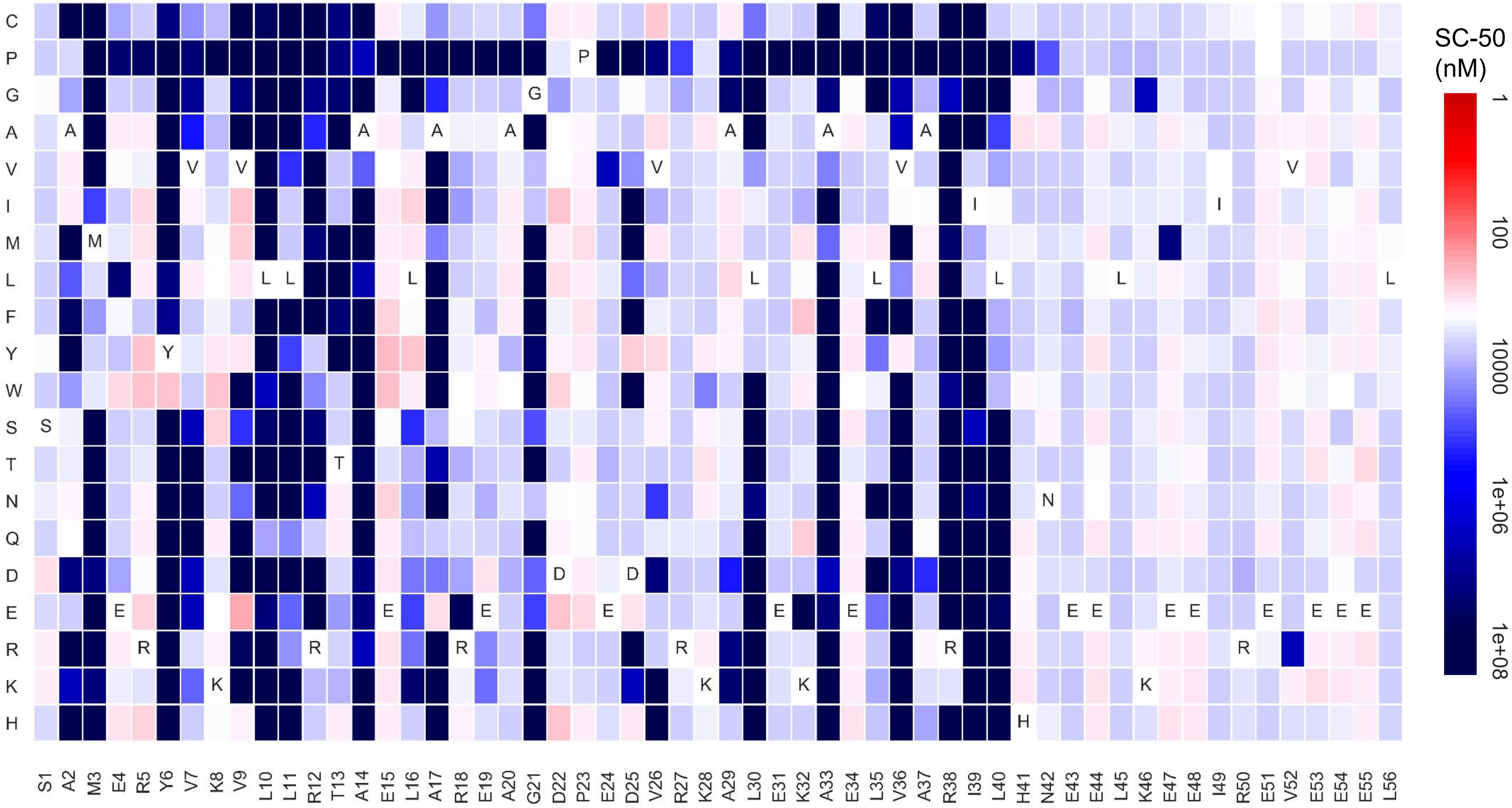

Minibinder 8

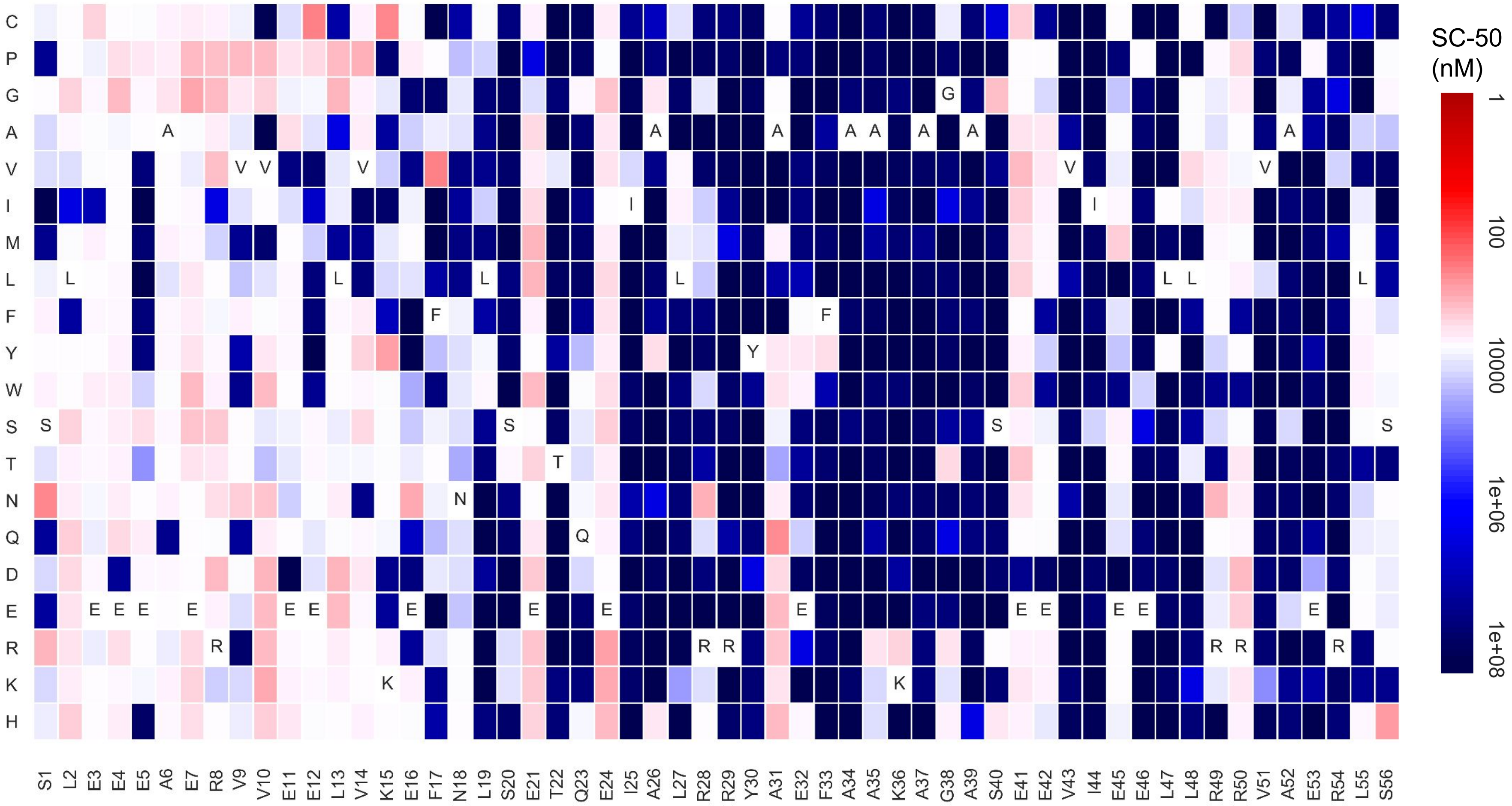

Minibinder 9

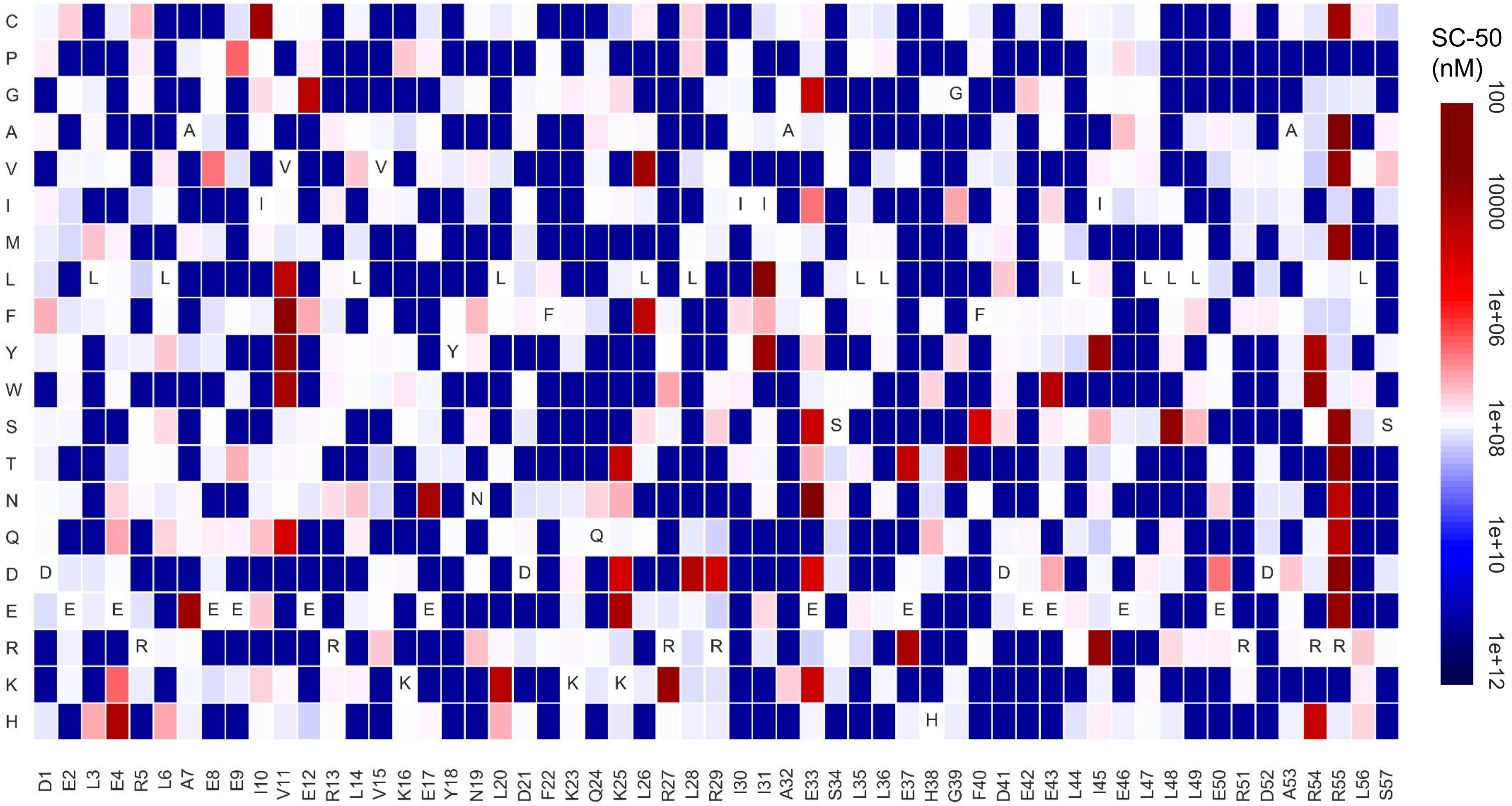

Minibinder 10

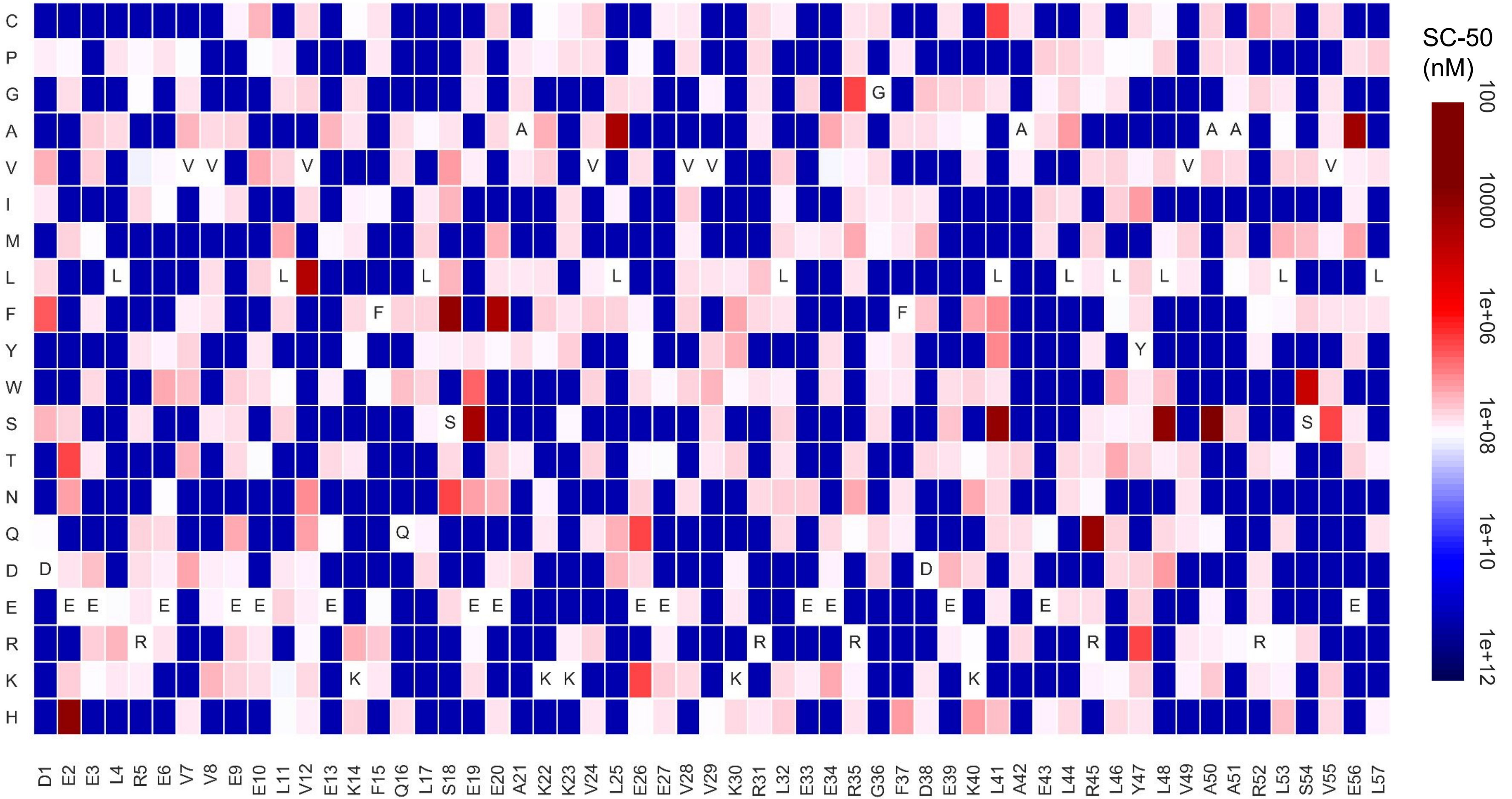

Minibinder 11

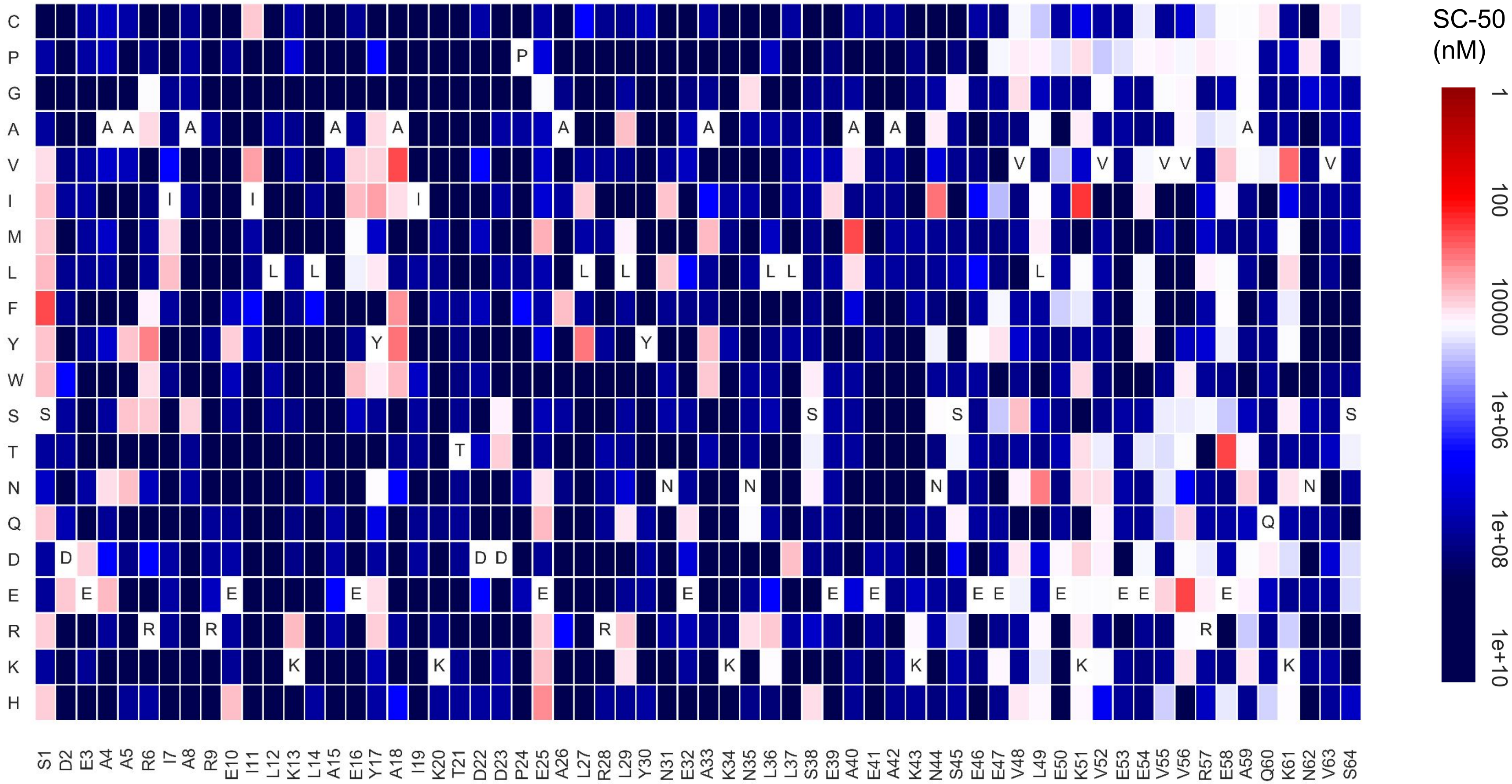

Full gel from Supplementary Fig. 1

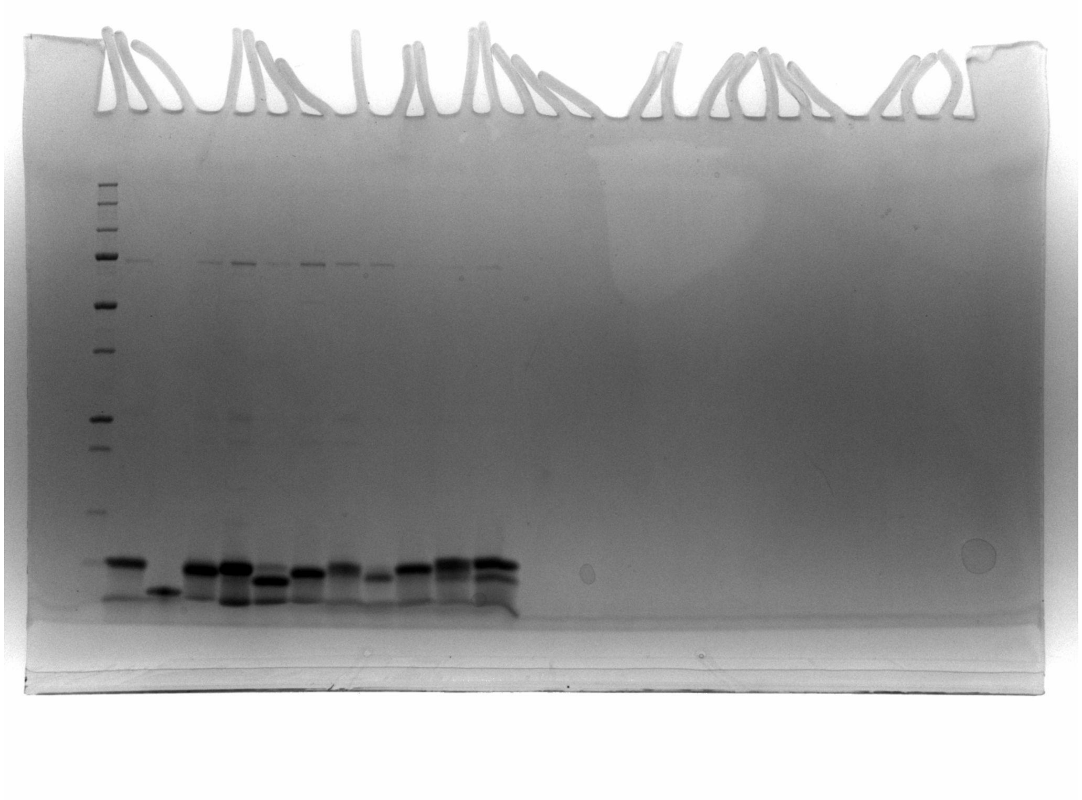

Full gel from Supplementary Fig. 3

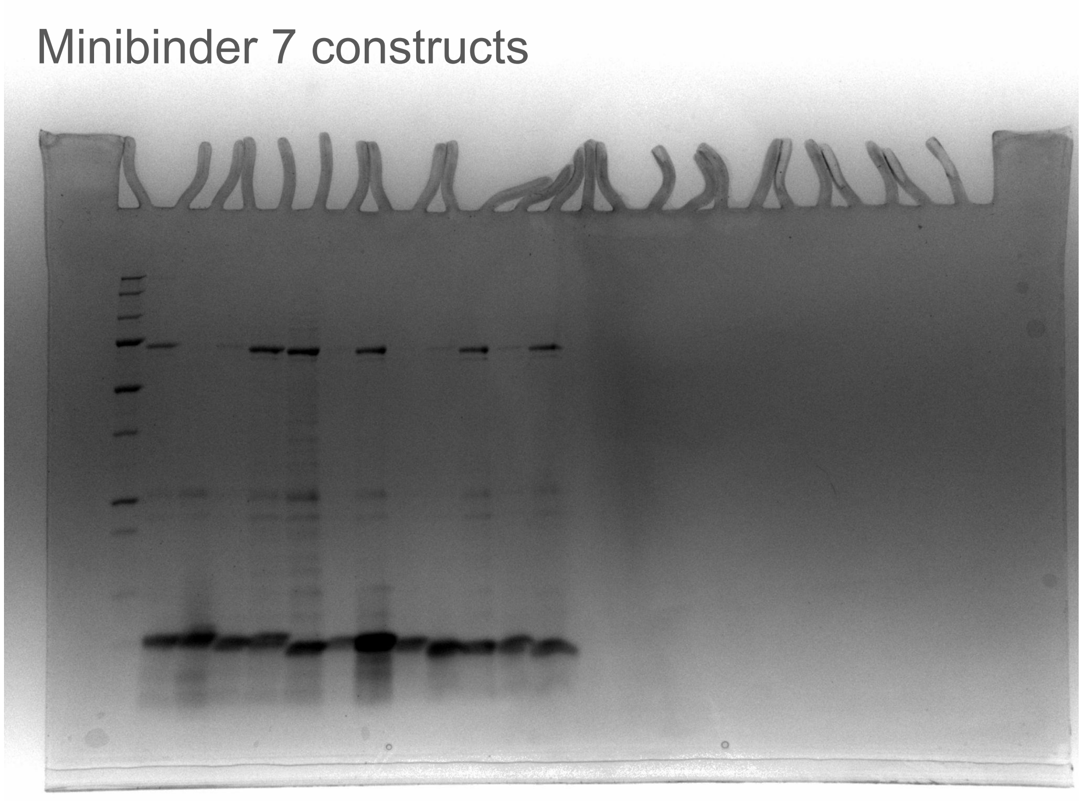

Full gel from Supplementary Fig. 3

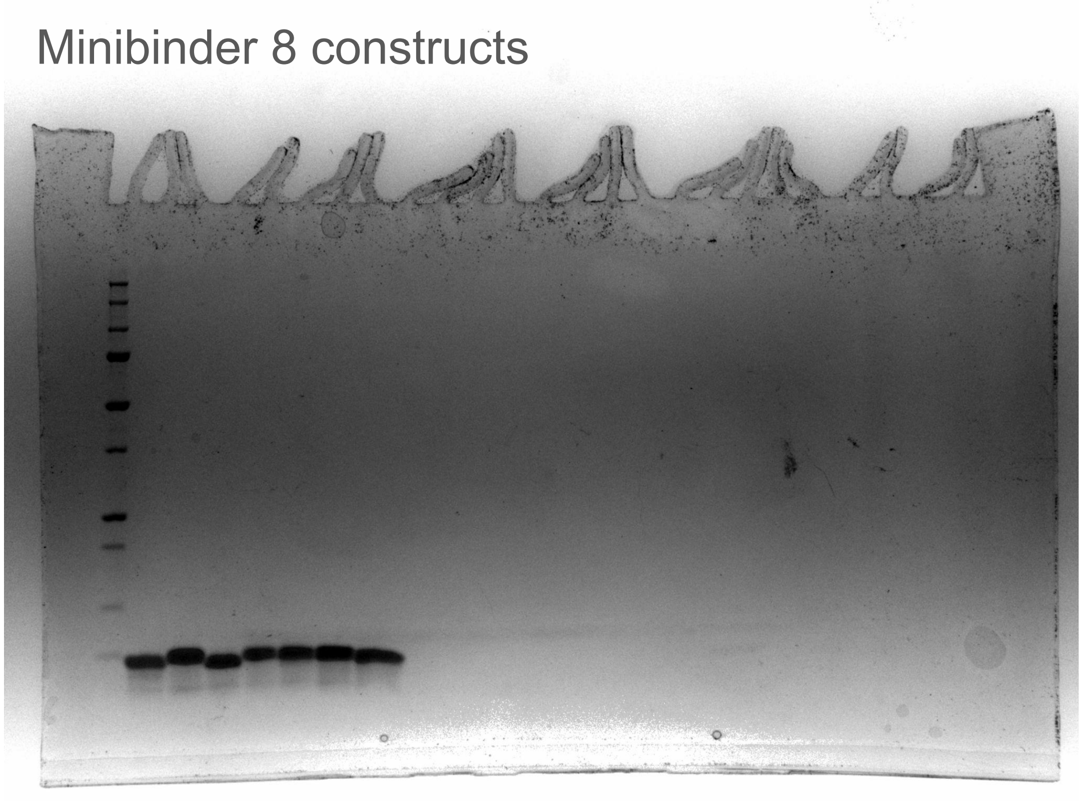

Full gel from Supplementary Fig. 9

TLR3

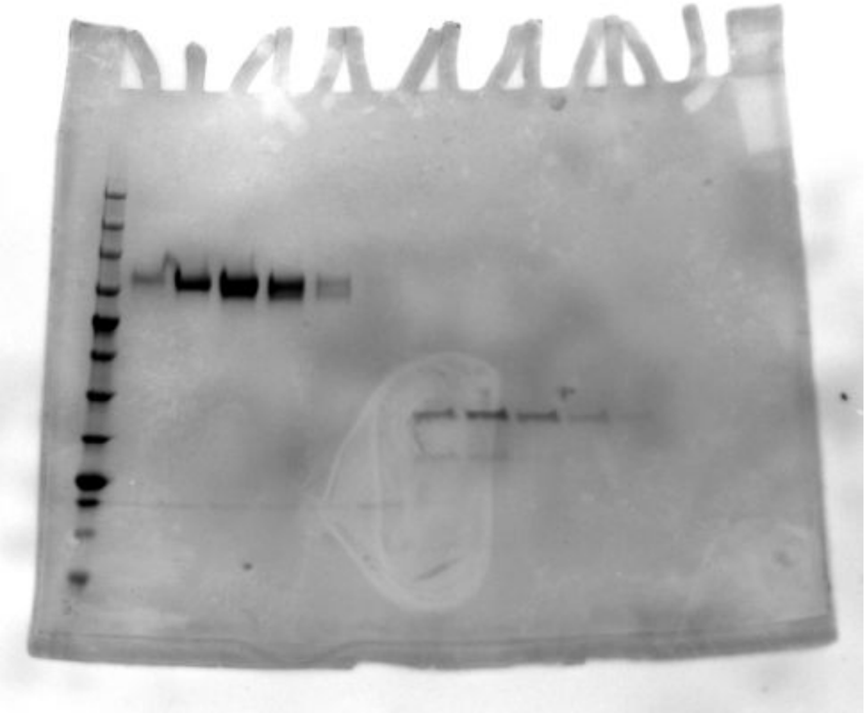

Full gel from Supplementary Fig. 9

TLR3 + 8.6 dimer

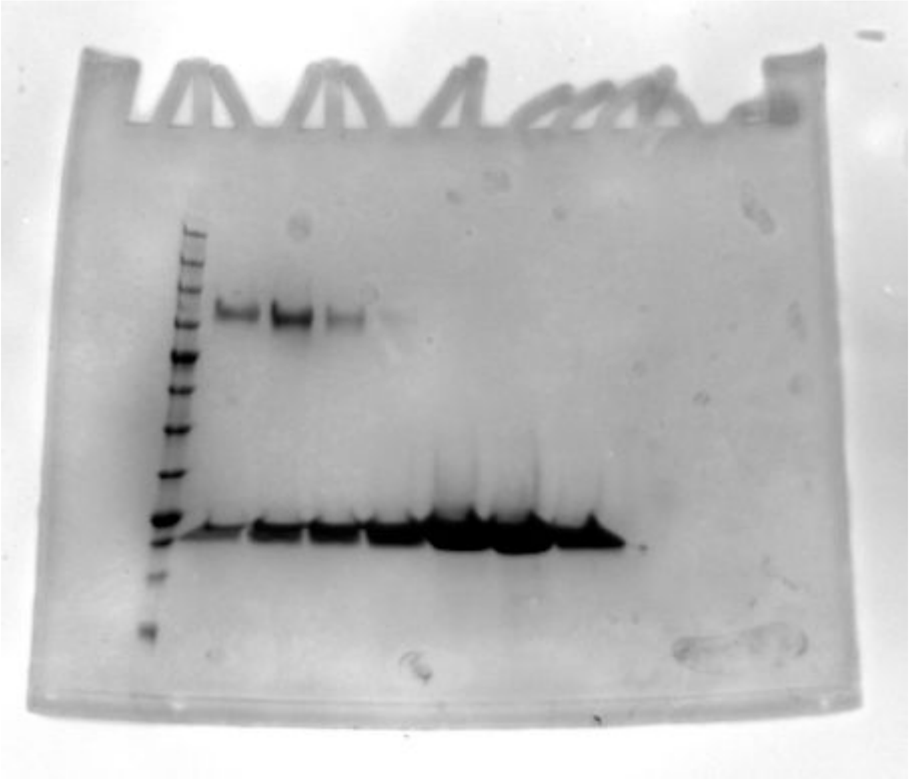

Full gel from Supplementary Fig. 9

TLR3 + 8.6 16GS tetravalent

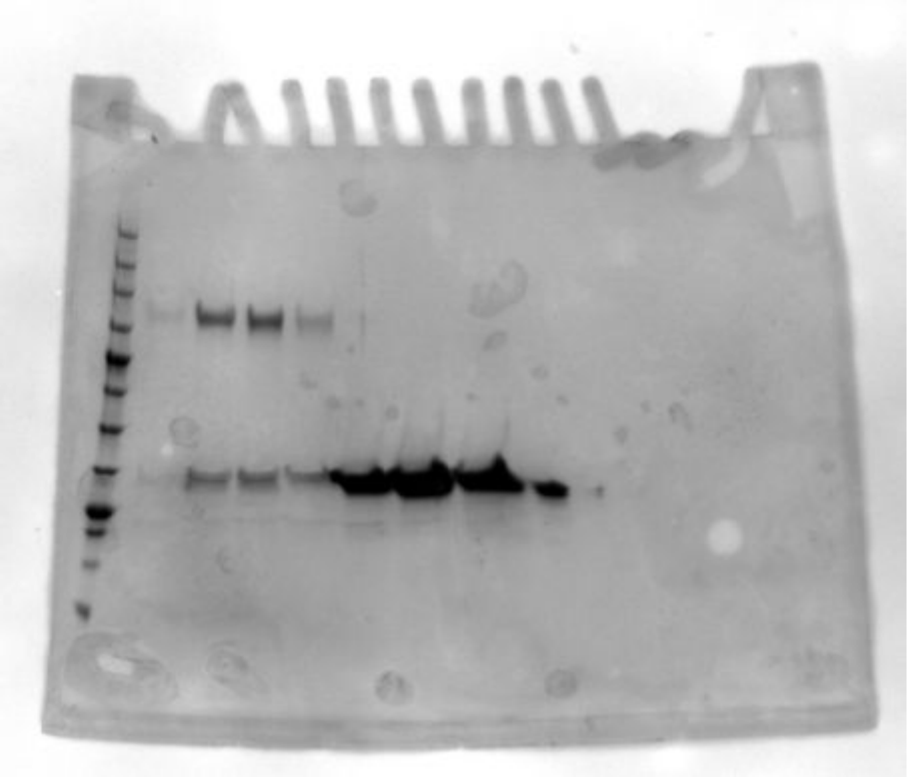

Supplement: Supplementary file 3 — Supplementary Data 1 [file 41467_2025_56369_MOESM3_ESM.pdf]
