# Supplementary material for: De novo design of protein minibinder agonists of TLR3
Source: Nat Commun. 2025 Jan 31;16:1234. doi: 10.1038/s41467-025-56369-w (PMC11785957; doi:10.1038/s41467-025-56369-w)
Supplement: Supplementary file 4 — Description of Additional Supplementary Files [file 41467_2025_56369_MOESM4_ESM.pdf]

Description of additional supplementary information

File name: Supplementary Data 1

Description: Minibinder heatmaps and Supplementary Figures full gel images.
